# Differences between *Trypanosoma brucei gambiense* Groups 1 and 2 in Their Resistance to Killing by Trypanolytic Factor 1

**DOI:** 10.1371/journal.pntd.0001287

**Published:** 2011-09-06

**Authors:** Paul Capewell, Nicola J. Veitch, C. Michael R. Turner, Jayne Raper, Matthew Berriman, Stephen L. Hajduk, Annette MacLeod

**Affiliations:** 1 College of Medical, Veterinary and Biological Sciences, Wellcome Trust Centre for Molecular Parasitology, University of Glasgow, Glasgow, United Kingdom; 2 College of Medical, Veterinary and Biological Sciences, University of Glasgow, Glasgow, United Kingdom; 3 Department of Microbiology, Langone School of Medicine, New York University, New York, New York, United States of America; 4 Wellcome Trust Sanger Institute, Hinxton, United Kingdom; 5 Department of Biochemistry and Molecular Biology, University of Georgia, Athens, Georgia, United States of America; Institute of Tropical Medicine, Belgium

## Abstract

**Background:**

The three sub-species of *Trypanosoma brucei* are important pathogens of sub-Saharan Africa. *T. b. brucei* is unable to infect humans due to sensitivity to trypanosome lytic factors (TLF) 1 and 2 found in human serum. *T. b. rhodesiense* and *T. b. gambiense* are able to resist lysis by TLF. There are two distinct sub-groups of *T. b. gambiense* that differ genetically and by human serum resistance phenotypes. Group 1 *T. b. gambiense* have an invariant phenotype whereas group 2 show variable resistance. Previous data indicated that group 1 *T. b. gambiense* are resistant to TLF-1 due in-part to reduced uptake of TLF-1 mediated by reduced expression of the TLF-1 receptor (the haptoglobin-hemoglobin receptor (*HpHbR)*) gene. Here we investigate if this is also true in group 2 parasites.

**Methodology:**

Isogenic resistant and sensitive group 2 *T. b. gambiense* were derived and compared to other *T. brucei* parasites. Both resistant and sensitive lines express the *HpHbR* gene at similar levels and internalized fluorescently labeled TLF-1 similar fashion to *T. b. brucei*. Both resistant and sensitive group 2, as well as group 1 *T. b. gambiense*, internalize recombinant APOL1, but only sensitive group 2 parasites are lysed.

**Conclusions:**

Our data indicate that, despite group 1 *T. b. gambiense* avoiding TLF-1, it is resistant to the main lytic component, APOL1. Similarly group 2 *T. b. gambiense* is innately resistant to APOL1, which could be based on the same mechanism. However, group 2 *T. b. gambiense* variably displays this phenotype and expression does not appear to correlate with a change in expression site or expression of *HpHbR*. Thus there are differences in the mechanism of human serum resistance between *T. b. gambiense* groups 1 and 2.

## Author Summary

The sub-species of *Trypanosoma brucei* are important pathogens of humans and animals in sub-Saharan Africa. *T. b. brucei* is not able to infect humans due to sensitivity to trypanosome lytic factors (TLF) containing the lytic protein apolipoprotein L1 (APOL1). *T. b. gambiense* is the most prevalent human infective sub-species, although there are two distinct sub-groups of *T. b. gambiense* that differ genetically and in terms of their human serum resistance phenotype stability. How they resist lysis by TLF is unknown. Previous data indicates that some group 1 *T. b. gambiense* parasites are able to avoid lysis due in part to reduced expression and activity of the gene encoding a TLF receptor (HpHbR). The authors showed that despite group 1 *T. b gambiense* displaying avoidance of some TLF particles, this sub-species group is resistant to the lytic protein of TLF, APOL1. This suggests that avoidance is not the complete story in this sub-species, as previously theorised. Group 2 *T. b. gambiense* is also innately resistant to APOL1 and there is no evidence of avoidance of TLF in resistant parasites. Group 2 *T. b. gambiense* variably displays the human serum resistance phenotype that does not appear to correlate with expression of *HbHpR*.

## Introduction

*Trypanosoma brucei* is an important parasite of sub-Saharan Africa. The species is commonly divided into three distinct sub-species that differ in geographical distribution, host range and the disease they cause. *T. b. brucei* is the most widespread form of the parasite, infecting a wide range of mammals, but is not able to infect humans because it is susceptible to trypanosome lytic factors (TLF) found in normal human serum [1]. *T. b. gambiense* and *T. b. rhodesiense* are, in contrast, resistant to lysis by human serum and so are able to infect humans. The West African trypanosome *T. b. gambiense* is the most prevalent form of the human-infective parasite and is responsible for greater than 90% of reported African sleeping sickness cases [2]. An important facet of African trypanosome biology is that the adaptive immune response of vertebrates is rendered largely ineffective due to the parasite's ability to cyclically evade the immune response by changing the variable surface glycoprotein (VSG) antigens on the cell surface [3], [4]. Several primates, including humans, therefore rely on innate immunity to prevent infection in the form of TLFs in serum that kill most species of trypanosome [5], [6], [7].

There are two distinct forms of TLF particle (TLF-1 and 2) in human serum. TLF-1 is a member of the high density lipoprotein (HDL) family of particles [8], [9] while TLF-2 is a related high molecular weight serum protein binding complex [10]. Considerable effort and much debate has been focused on determining the cytotoxic component of these TLFs, predominantly focusing on TLF-1 due to the difficulty of obtaining active TLF-2 [10]. Investigation has centered on two primate-specific proteins found in TLF; haptoglobin-related protein (HPR) and apolipoprotein L1 (APOL1). While there has been some controversy concerning the roles of these two proteins, the current consensus is that both proteins are necessary for optimal lysis and that HPR and APOL1 have complementary roles [11], [12]. HPR is the ligand that facilitates uptake of TLF-1 via a parasite haptoglobin-hemoglobin receptor (HpHbR) [13], [14] and it has also been shown to have some trypanosome specific toxicity [15], possibly due to an un-cleaved signal peptide that affects membrane fluidity [16]. After internalization, the TLF-1 particle is then trafficked to the lysosome where APOL1 is activated by a pH-mediated conformational change to form pores in the lysosome membrane. This leads to perturbation of the osmotic balance of the organelle and subsequent lysis [17], [18]. As both HPR and APOL1 are found in TLF-2, it is likely that the lysis method of this particle involves both of these proteins, although the method of entry for TLF-2 does not directly involve the HpHbR receptor [13], [19].

The two human infective trypanosome sub-species have evolved counter-measures to overcome the innate defense factors. Understanding the mechanism of how these parasites resist lysis may lead to the development of new treatment strategies. For example, the resistance mechanism for *T. b. rhodesiense* is dependent upon the expression of a serum resistance associated (*SRA*) gene. This has led to new diagnostic techniques [20] and therapeutic possibilities targeting *SRA* [21], [22]. The *SRA* gene has not been found in any *T. b. gambiense* isolates however [23], [24], [25], so an alternate and still unknown mechanism of human serum resistance exists in this subspecies.

A complicating factor is that *T. b. gambiense* possesses two distinct “groups” that differ in genotype and phenotype. Isoenzyme and molecular data show that group 1 and group 2 *T. b. gambiense* populations are reliably distinguishable from each other, and that group 2 is more akin to *T. b. brucei* than group 1 *T. b. gambiense* [26], [27], [28], [29], [30], [31], [32], [33], [34], [35]. In group 1 *T. b. gambiense*, human serum resistance is a stable trait [31], [33] and it has been suggested that a reduced uptake of TLF-1 may be partially responsible for resistance. These parasites exhibit reduced expression of *HbHpR*, which may contribute to their ability to infect humans by avoiding TLF-1 [36]. In addition, mutant or transgenic *T. b. brucei* lines with reduced expression of the *HpHbR* receptor gene are resistant to TLF-1 particles [13], [36], although these lines are still lysed by normal human serum and TLF-2. This raises the possibility that group 1 *T. b. gambiense* parasites are sensitive to APOL1 and that they avoid lysis by failing to take up APOL1 whether it is in TLF-1 or 2. However, uptake of TLF-2 in these parasites has not so far been examined. In contrast, group 2 *T. b. gambiense* exhibit a variable human serum resistance phenotype in a manner superficially similar to *T. b. rhodesiense*, raising the question: do groups 1 and 2 *T. b. gambiense* share a common mechanism of human serum resistance? To address this question we have compared the properties of the human serum resistance phenotypes of group 1 and group 2 *T. b. gambiense* by examining the effects and uptake of both TLF-1 and recombinant APOL1 in these parasites.

## Methods

### *T. b. gambiense*, *T. b. brucei* and *T. b. rhodesiense* cell lines

The human serum resistant group 2 *T. b. gambiense* strain STIB386 was originally isolated in 1978 from an infected patient in the Ivory Coast. ELIANE is a group 1 *T. b. gambiense* strain isolated from a human infected while in Côte d'Ivoire in 1952 [37]. STIB247 is a human serum sensitive *T. b. brucei* clone, first isolated from a hartebeest in Serengeti in 1971 and the *T. b. rhodesiense* strain Baganzi was isolated from a human in South-Eastern Uganda in 1990. All lines were maintained *in vitro* in HMI9 medium [38] supplemented by 1.5 mM glucose, 1 mM methyl cellulose, 250 µM adenosine, 150 µM guanosine and 20% serum (fetal bovine serum (FBS) for *T. b. brucei* and sensitive group 2 *T. b. gambiense* or human serum for the other strains) . All lines were regularly assayed for human serum sensitivity/resistance.

### Human serum resistance & APOL1 assays

Trypanosomes were diluted to 10^6^ per ml in modified HMI9 and incubated in 25% human serum or FBS in a 1 ml volume in a standard 24 well plate. The numbers of cells in each well were counted with a hemocytometer for the zero time point. The cells were incubated with 5% CO_2_ at 37°C and the number of viable motile trypanosomes in each well at 6 hours was quantified by microscopy using a hemocytometer and the percentage of viable cells calculated compared to time zero.

For the APOL1 lysis assays, a dilution series of recombinant APOL1 (5–50 µg/ml) was formulated that and made up to equal volumes with protein free buffer (0.2 M acetic acid and 0.05% Tween20). A control containing an equal volume of buffer was also prepared. Each protein volume and the control were aliquoted into different wells and the cells incubated with 5% CO_2_ at 37°C in HMI9 medium containing 25% FBS. The number of viable motile trypanosomes in each well was recorded at 24 hours and compared to the control wells containing no APOL1 to determine percentage survival. In each assay, cells were incubated in 25% normal human serum as a positive control.

### Generation of recombinant APOL1

An Invitrogen Gateway®-compatible entry vector containing the *APOL1* open reading frame (ORF) (Genecopoeia Inc, USA) was used in conjunction with the Invitrogen Gateway® expression system. The *APOL1* was cloned into pDest17 destination vector containing an N-terminal 6×His-tag and transformed into BL21-AI competent *E. coli*. Protein expression was induced using 0.1% L-Arabinose for 16 hours at 37°C. Cells were lysed with guanidinium lysis buffer pH 7.8 (6 M Guanidine Hydrochloride, 0.02 M Sodium Phosphate, 0.5 M NaCl) for 5 minutes and the cellular debris removed with a 0.2 µm filter (Sartorius). The cell lysate was bound to Ni-NTA beads (Invitrogen) for one hour at pH 7.8 and then washed twice with wash buffer (8 M Urea, 0.02 M Sodium Phosphate, 0.5 M NaCl) at pH 7.8, followed by two washes at pH 6 and two washes at pH 5.8. Finally, bound protein was eluted with wash buffer at pH 4. The eluate was dialyzed overnight against 0.2 M acetic acid and 0.05% Tween20 and concentrated using 10,000MW Vivaspin columns (Sartorius). Protein purity was estimated using a Nanodrop® spectrometer (Nanodrop) and SDS-PAGE. A Western blot using an antibody raised against an APOL1 peptide (Sigma-Aldrich) was used to check that the single band present in the purified preparation was APOL1. Activity of the recombinant protein was determined by its ability to lyse *T. b. brucei*. The specific activity of the protein was estimated using a previously defined method using the amount protein needed to lyse 50% of the cell within 2 hours [17]. This value was determined to be 0.143 units/mg, which is comparable to purified native APOL1, although much lower than an intact TLF-1 particle that has a specific activity of 83.3 units/mg [39].

### Fluorescence microscopy of TLF-1 uptake

TLF-1 was prepared as previously described [10] and the purity of preparation was verified by both western blot and SDS PAGE using an APOL1 specific antibody (Sigma-Aldrich). TLF-1 was labeled with AlexaFluor®488 (Molecular Probes, Invitrogen) using the manufacturer's instructions. In control experiments, bovine HDL of a density comparable to TLF-1 [10] was also AlexaFluor tagged. Trypanosomes were re-suspended in serum-free HMI9 medium at a concentration of 10^6^ cells/ml and incubated in 10 µg/ml of Lysotracker® (Invitrogen) and 5 µg/ml of the purified AlexaFluor tagged human TLF-1 [10]. The cells were incubated at 37°C for 30 minutes, 1, 2 and 4 hours. At each time point, cells were washed once in serum-free HMI9 medium and fixed by immersion in chilled 2.5% gluteraldhyde (Sigma-Aldrich) in phosphate buffered saline for 5 minutes. The cells were re-suspended in 50% glycerol, 0.1% DAPI, 2.5% DABCO in phosphate buffered saline (PBS) and spread onto lysine-coated slides which were then protected with cover-slips sealed using ethyl acetate.

Slides were imaged using the Deltavision Core system and SoftWorx package (Applied Precision) with standard filter sets (DAPI/FITC/Texas-Red and Light transmission). Approximately 30 serial sections through each trypanosome were taken for each filter. The images were composited and the brightness, contrast and color levels normalized between samples and exposures using the ImageJ software package (US National Institute of Health). Approximately thirty trypanosomes were imaged per time point to give an indication of the uptake in the population. The Pearson's correlation co-efficient between the TLF and Lysotracker® fields was calculated using the Pearson-Spearman Correlation (PSC) Plug-in for ImageJ (http://www.cpib.ac.uk/~afrench/coloc.html).

### Fluorescence microscopy of APOL1 uptake

Trypanosomes were re-suspended in HMI9 medium containing 20% fetal bovine serum at a concentration of 10^6^ cells/ml and incubated in 10 µg/ml of Lysotracker® (Invitrogen) and purified recombinant APOL1 (5–50 µg/ml). The cells were incubated at 37°C for 4 hours. After this period, cells were washed once in serum-free HMI9 medium and fixed by immersion in chilled 2.5% gluteraldhyde (Sigma-Aldrich) in phosphate buffered saline for 5 minutes. The cells were washed once more in chilled PBS and then re-suspended in PBS with an AlexaFluor®488 His-Tag antibody (Molecular Probes, Invitrogen). The cells were gently agitated for 1 hour and then washed twice with chilled PBS and spread onto slides as described above.

### Real-time PCR

Total trypanosome RNA was extracted from approximately 5×10^6^ cells using a Qiagen RNAeasy® mini kit following the manufacturer's instructions. The RNA was subjected to three DNase I (Invitrogen) digests to remove all genomic contamination. Omniscript® RT Kit (Qiagen) was used to generate cDNA from 1 µg of total RNA as per the manufacturer's instructions. Real-time-PCR was performed using cDNA from an equivalent of 50 ng of total RNA, 3 µM forward and reverse primers (HpHbR_RT_F & HpHbR_RT_R, Table 1) and 12.5 µl of SYBR green PCR master mix (Applied Biosystems) to a final volume of 25 µl. Real-time PCR conditions were: one cycle of 50°C for 2 mins, 95°C for 10 mins, followed by 40 cycles of 95°C for 15 s, 60°C for 1 min. The relative amounts of specific cDNA between samples were calculated using C_T_ methodology [40] calculated with the Applied Biosystems SDS v1.4 software. The endogenous control gene was *GPI8* using primers *GPI8*F and *GPI8*R primers (Table 1). Four biological replicates were performed for each parasite line. All primers were designed using the Primer3 software [41]. Mean levels of expression were compared using 1-way ANOVA and Tukey's post hoc test.

**Table 1:** PCR primers.

Primer	Sequence 5′-3′
HpHbHpr RT F	GCCCTATGCTTATGCACATGATC
HpHbHpr RT R	ACCTCCGCCAGAGAAAATCTC
GPI8F	ATACAACGAATGCGCTGGCC
GPI8R	ACCTCCGCCAGAGAAAATCTC
HpHbHpr A F	ACAAAGTGGCAGGTGCGTTG
HpHbHpr A R	ATT TTC GAT CGG GTT CCC AT
HpHbHpr B F	ATG GGA ACC CGA TCG AAA AT
HpHbHpr B R	AATCAGTTTTTTAGGGCGGC
ESAG6 F	CCGGAATTCGCTATTATTAGAACAGTTTCT
ESAG6 R	GTGTTAAAATATATC
ESAG7 F	CCGGAATTCGCTATTATTAGAACAGTTTCT
ESAG7 R	GCTCTAGACATCACTGCATTTTTTGCTTC
SRA F	GACAACAAGTACCTTGGCGC
SRA R	CAGCAACCATATTCAGAGCC
TgSGP F	TCACGGCCATCAGACGGAGA
TgSGP R	GCCATCGTGCTTGCCGCTC
JS2 F	GATTGGCGCAACAACTTTCACATACG
JS2 R	CCCTTTCTTCCTTGGCCATTGTTTTACTAT

### Sequencing and PCR

To test for the presence/absence of *SRA* and *T. b. gambiense* specific glycoprotein (*TgSGP*) [42], primer sets *SRA*-F with *SRA*-R and *TgSGP*-F with *TgSGP*-R [20], [37], [43] were used to amplify from prepared genomic DNA. The *SRA* gene was amplified using the *SRA* primer set under the following conditions with *Taq* polymerase for 30 cycles; 95°C for 50 seconds, 55°C for 50 seconds and 65°C for 60 seconds while the *TgSGP* primers were amplified with the conditions for 30 cycles; 95°C for 50 seconds, 60°C for 50 seconds and 65°C for 90 seconds. Primer sequences are given in Table 1.

The *HpHbR* ORF and 1000 bp downstream sequence containing the 3′ UTR were amplified from genomic DNA by PCR using the following conditions with *PFU* polymerase for 30 cycles; 95°C for 50 seconds, 55°C for 50 seconds and 65°C for 120 seconds.

PCR products were ligated into the TOPO PCR 2.1 plasmid (Invitrogen) and amplified using TOP10 (Invitrogen) competent cells as per the manufacturer's instructions. The plasmid was purified using a Qiagen miniprep kit and sent for DNA sequencing at DNA Sequencing & Services (MRCPPU, University of Dundee, www.dnaseq.co.uk) using Applied Biosystems Big-Dye Ver 3.1 chemistry on an Applied Biosystems model 3730 automated capillary DNA sequencer.

The variable regions of the transferrin receptor genes (*ESAG6* and *ESAG7*) [44], [45] were amplified from cDNA prepared as previously described. Primer sets *ESAG6*_F & *ESAG6*_R and *ESAG7*_F & *ESAG7*_R (Table 1) were used under the following PCR conditions with PFU polymerase for 30 cycles; 95°C for 50 seconds, 55°C for 50 seconds and 65°C for 60 seconds. PCR products were sequenced as previously described.

## Results

### Human serum resistance phenotypes of different sub-species

**Figure 1:**
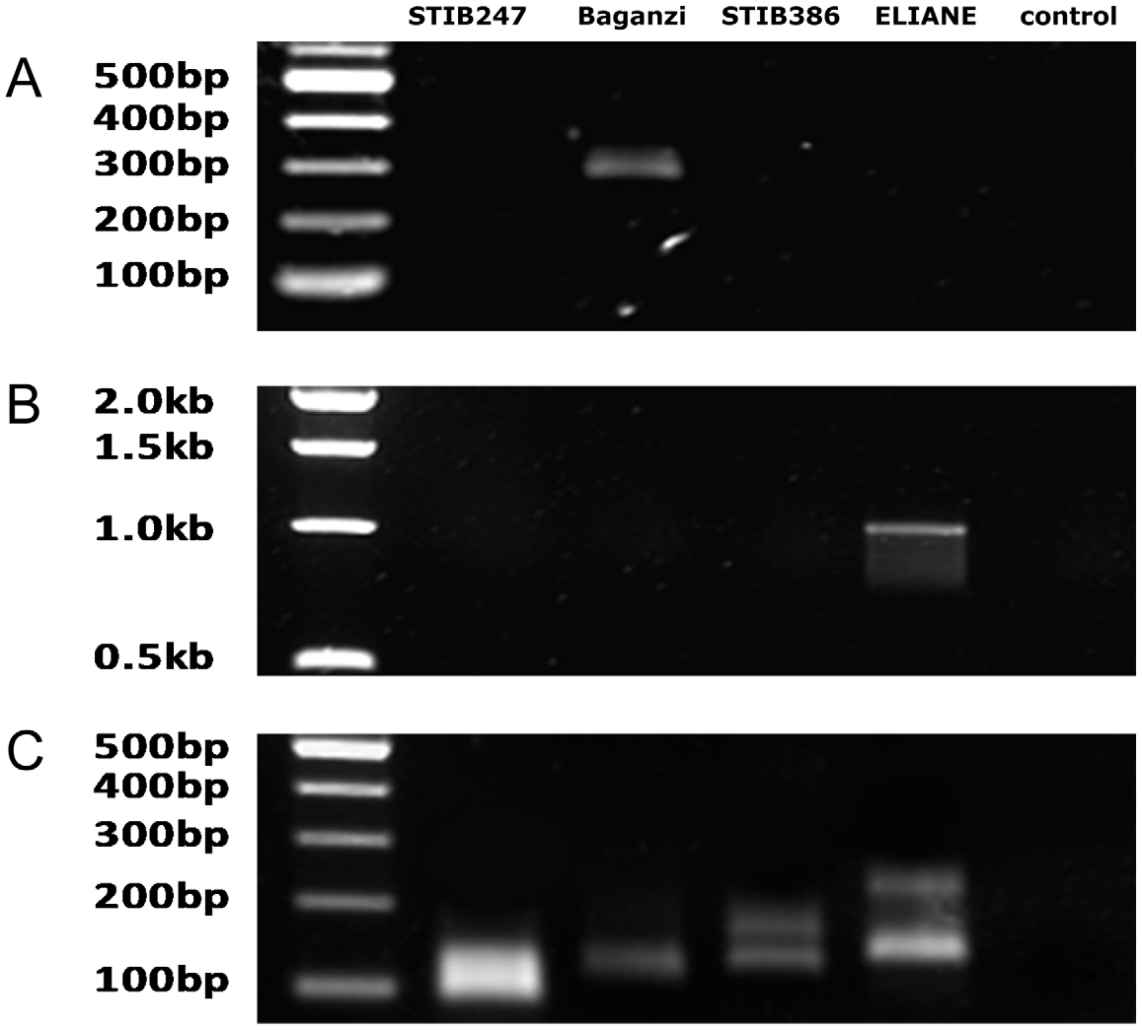
Identification of sub-species of *T. brucei* lines. PCR amplification of (A) the *SRA* gene, (B) the *TgSGP* gene and (C) microsatellite marker JS2 as a template control. Track 1 = ladder, track 2 = *T. b. brucei* STIB247, track 3 = *T. b. rhodesiense* Baganzi; track 4 = group 2 *T. b. gambiense* STIB386, track 5 = group 1 *T. b. gambiense* ELIANE and track 6 = no template control.

**Figure 2:**
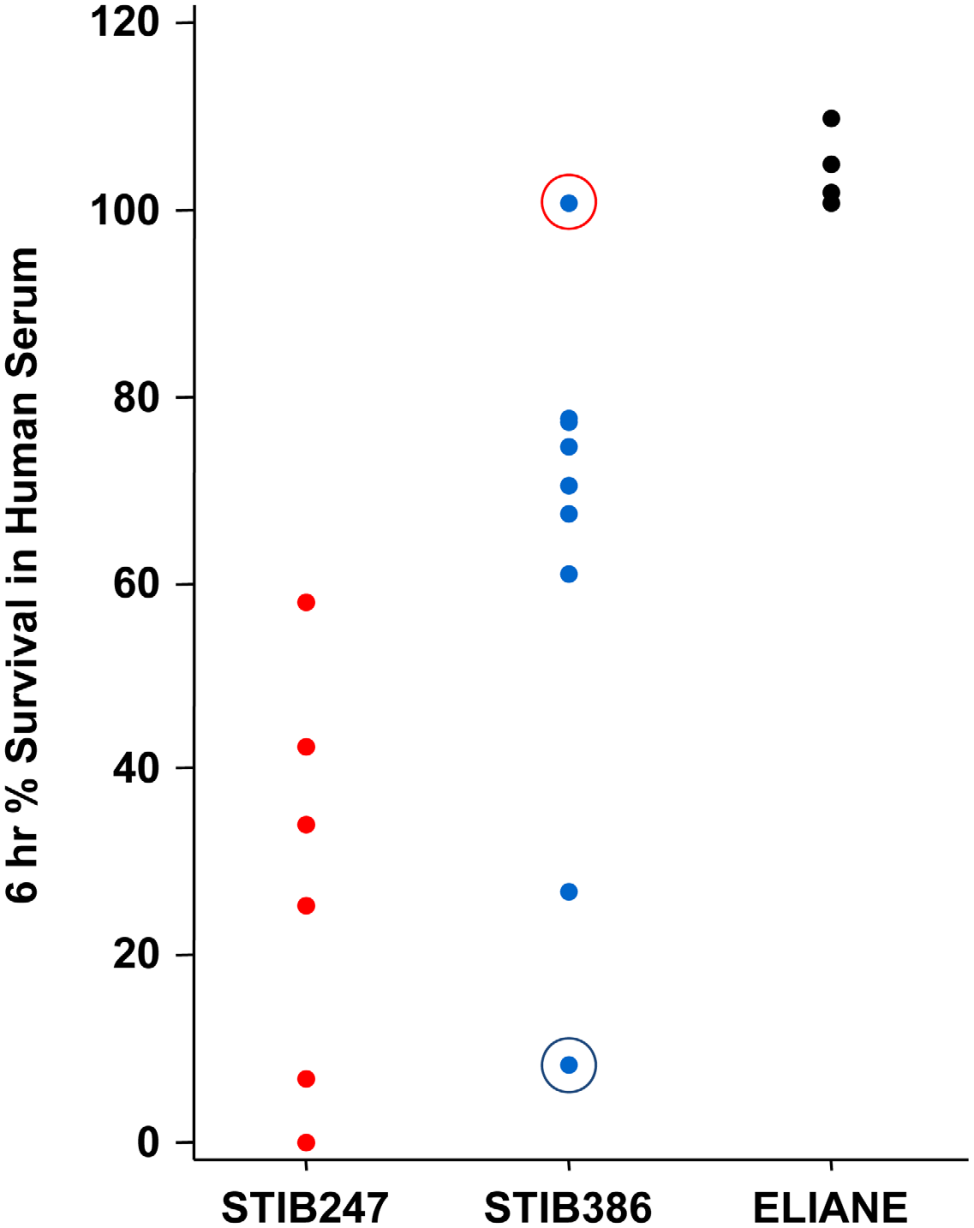
Human serum resistance phenotype. The survival of parasites exposed to 25% lytic human serum for 6 hours as a percentage of the starting cell count. Each value is the mean result from a separate experiment (for ELIANE, n = 4). The two isolate populations that were selected to create *T. b. gambiense* STIB386S (circled in blue) and *T. b. gambiense* STIB386R (circled in red) are indicated.

To investigate human serum resistance/sensitivity in the two different groups of *T. b. gambiense* STIB386 (group 2 *T. b. gambiense*) and ELIANE (group 1 *T. b. gambiense*) were compared with each other and with representative isolates of each of the other sub-species; STIB247 (*T. b. brucei*) and Baganzi (*T. b. rhodesiense*). Strains were cultured and examined for the presence/absence of the sub-species specific genes *SRA* (*T. b. rhodesiense* specific [46]) and *TgSGP* (group 1 *T. b. gambiense* specific [37]) by PCR amplification in order to verify their sub-species classification (Figure 1). The *SRA* gene was found to be present in the *T. b. rhodesiense* Baganzi strain and the *TgSGP* gene was present in the group 1 *T. b. gambiense* ELIANE strain, confirming their sub-species status. The strains *T. b. brucei* STIB247 and group 2 *T. b. gambiense* STIB386 do not possess either gene (Figure 1). The human serum resistance phenotype for the *T. b. brucei* and *T. b. gambiense* strains were established by monitoring survival in the presence of human serum over 6 hours (Figure 2). The group 1 *T. b. gambiense* strain ELIANE was always resistant to human serum with 100% survival and the *T. b. brucei* STIB247 was always sensitive with a mean survival of less than 25% (Figure 2). These results confirm the sub-species classification of each of the strains used in the subsequent experiments, (as previously described for STIB247, ELIANE and STIB386 [46]). The marked variation in human serum resistance phenotype previously described in *T. b. rhodesiense* is also seen in group 2 *T. b. gambiense*. By culturing the strain *T. b. gambiense* STIB386 in the presence of 10% human serum in an *in vitro* system we selected a population of human serum resistant parasites (STIB386R; Figure 2). Several clonal populations of the original STIB386 clone gave a range of human serum resistance phenotypes of which one sensitive line was chosen that was consistently sensitive to human serum in continuous culture without human serum (STIB386S; Figure 2). In order to demonstrate that the lines were indeed isogenic, a series of four microsatellite markers that are used routinely to genotype isolates, Tb5/4, Tb2/20, Tb2/10 and Tb3/1, were amplified using the conditions previously described [47], giving identical genotypes (unpublished). Thus isogenic lines of sensitive and resistant parasites were available for comparison.

### Bloodstream expression site analysis

Previous studies indicate that the switching of resistance in *T. b. rhodesiense* strains correlated with a change in the variable antigen type (VAT) [23], [24], [48], [49]. In order to determine if a change in *VSG* expression site (ES) has occurred, the *ESAG6* and *ESAG7* genes are routinely used as ES markers as they contain polymorphisms that are associated with each expression site [45], [50], [51], [52]. However, while these markers are useful for *T. b. brucei* it is possible that the group 2 *T. b. gambiense* strain used in our study has a reduced repertoire of *ESAG6* and *ESAG7* variants compared to *T. b. brucei*, in a similar manner to group 1 *T. b. gambiense*, which has been shown to possess a smaller repertoire of *ESAG*6 and *ESAG7* than other *T. brucei* sub-species [53]. The *ESAG*s of group 2 *T. b. gambiense* have not been studied in detail, although an estimate of the variation of *ESAG6* and *ESAG7* can be determined from the available genome sequence for this strain (Berriman, unpublished). The genome sequence for the other sub-species were also available for analysis: the *T. b. brucei* strains STIB247 (Berriman, unpublished), the genome reference TREU927 [54] and the group 1 *T. b. gambiense* strain DAL972 [55]. Sequence reads were aligned to the published variants of *ESAG6* and *ESAG7* [53], [54], [56], [57], [58]. Sequence reads from STIB247, TREU927 and STIB386 aligned to 21, 22 and 22 unique *ESAG6* and 21, 22 and 21 unique *ESAG7* sequences, respectively (Figure S1). However, the group 1 *T. b. gambiense* strain DAL972 [55] sequencing analysis only showed aligned reads for eight unique *ESAG6* sequences and nine unique *ESAG7* sequences. This parallels previously published data revealing that group 1 *T. b. gambiense* have a less variable *ESAG* complement than *T. b. brucei* [53]. Alternatively, it may be the case that group 1 *T. b. gambiense* has an *ESAG* complement containing variants that differ from the *T. brucei ESAG6* and *ESAG7* sequences previously published. Whichever hypothesis is correct, both suggest that the group 2 *T. b. gambiense* strain STIB386 possess a *T. b. brucei*-like rather than a group 1-like *ESAG* repertoire. This is consistent with other studies suggesting that group 2 *T. b. gambiense* are more similar to *T. b. brucei* than group 1 *T. b. gambiense* [59], [60] including the possession of metacyclic *ESAG*s found in *T. b. brucei* and *T. b. rhodesiense*, but not group 1 *T. b. gambiense* [57]. These results suggest that *ESAG6* and *ESAG7* variants are suitable ES markers.

In order to determine if a switch in ES is associated with the human serum resistance phenotype in STIB386, we examined the ES used in both sensitive and resistant lines using *ESAG6* and *ESAG7* as ES markers. RNA from both sensitive and resistant isogenic lines was extracted and used to generated cDNA. The hyper-variable regions of *ESAG6* and *ESAG7* genes were then amplified by PCR and sequenced. No sequence differences were detected between the hyper-variable regions of *ESAG6* and *ESAG7* in *T. b. gambiense* STIB386S and STIB386R lines. Multiple clones from the PCR reactions were sequenced confirming that the dominant *ESAGs* from the active ES were detected (Figure S2). These results are consistent with the view that both isogenic lines are using the same dominant ES, suggesting that human serum resistance in group 2 *T. b. gambiense* is not associated with ES switching.

### *HpHbR* expression

**Figure 3:**
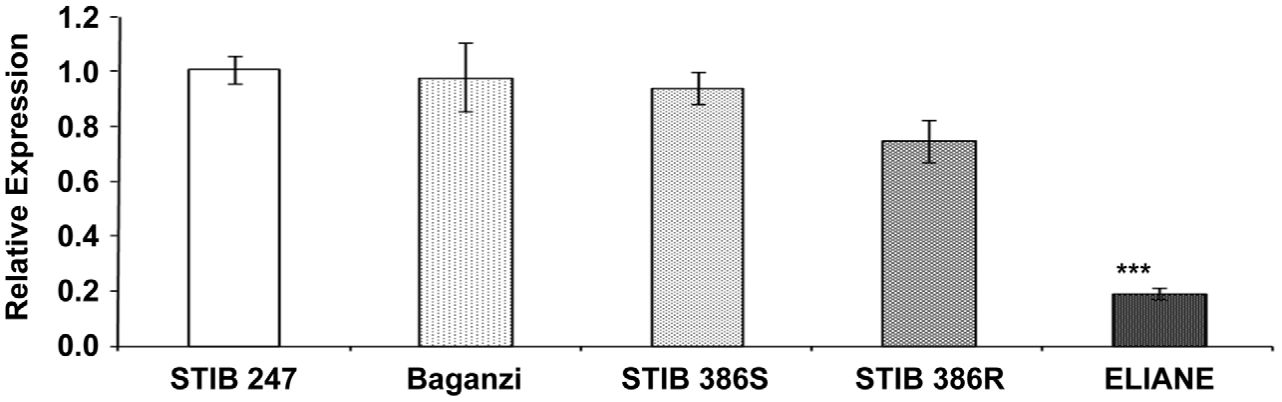
Relative *HpHbR* expression. The relative abundance of transcripts for the *HpHbR* in each cell line relative to *GPI8* as internal control. All samples were normalized against the mean *T. b. brucei* strain STIB247 C_T_ value (n = 4 for each line and standard error of the mean C_T_ values is shown). Statistically significant value (P<0.01) is indicated with an asterisk.

Recent evidence indicates that *T. b. gambiense* group 1 parasites do not take up TLF-1 due to reduced expression and function of HpHbR [36]. A reduction of expression of this gene in *T. b. brucei* by RNAi or gene knockout confers TLF-1 resistance [13], [36]. Do group 2 *T. b. gambiense* parasites employ a similar mechanism? To address this question, the expression of the *HpHbR* gene was examined in the different lines by measuring the relative amounts of transcript for the gene using quantitative real-time PCR. The *T. b. brucei* line (STIB247), isogenic lines of *T. b. gambiense* group 2 (STIB386S and STIB386R) and *T. b. rhodesiense* (Baganzi) strains all expressed similar levels of transcript for the *HpHbR* gene relative to a *GPI8* endogenous control (Figure 3). Expression levels of the *GPI8* control did not show much variation across the different isolates. The STIB386R strain showed a slight decrease in mean level of expression of the gene compared to STIB386S but this was not statistically significant using a one-way ANOVA (F_7_ = 4.03, p = 0.091). However, the group 1 *T. b. gambiense* strain expressed a five-fold lower mean transcript level compared to the other parasite lines and comparing expression across all five strains identified a significant difference (F_18_ = 36.51, p<0.01). Post-hoc Tukey's tests indicated the group 1 *T. b. gambiense* strain ELIANE to be different to all other lines whilst all comparisons between the other four lines were not significant.

### TLF-1 uptake and localization

**Figure 4:**
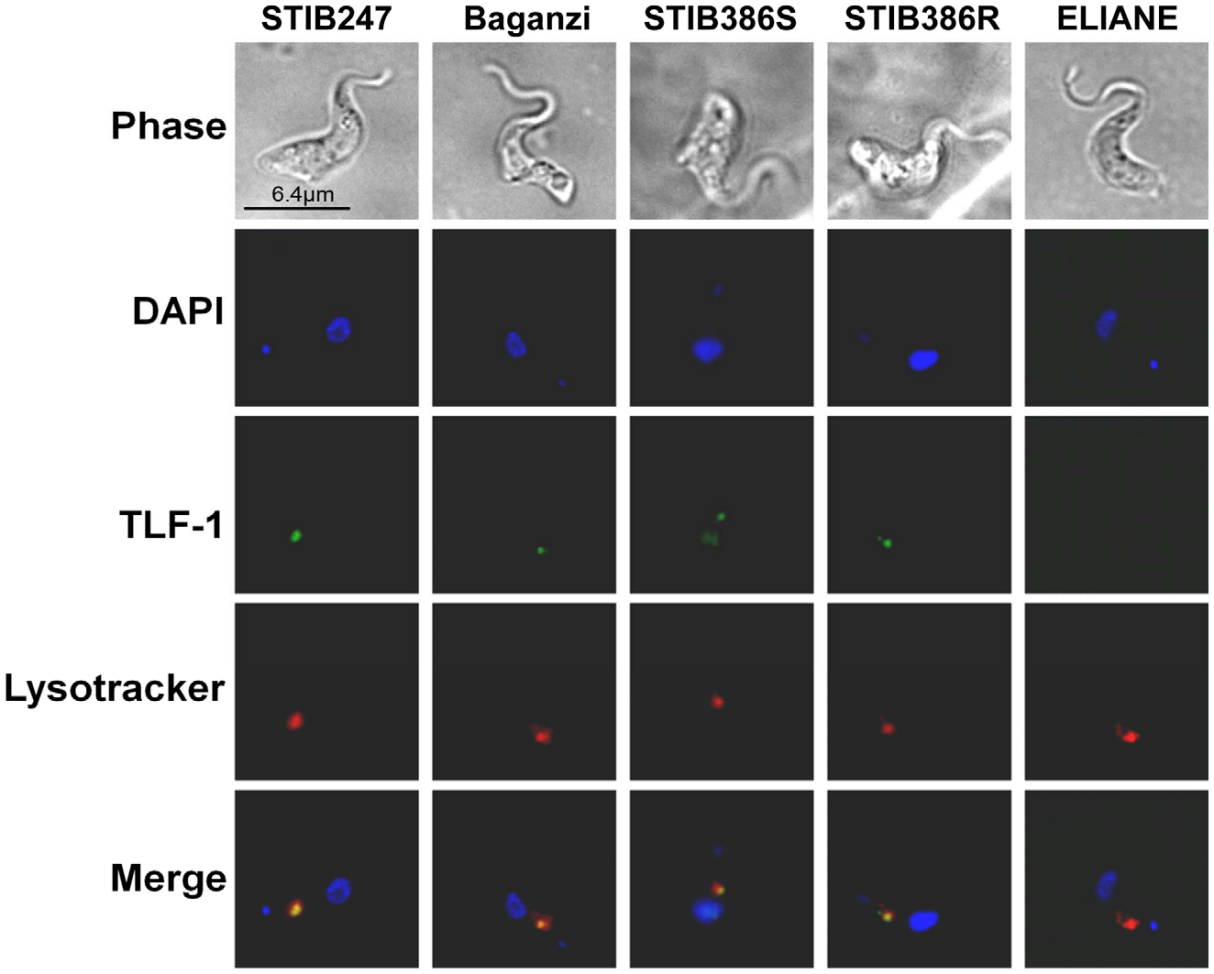
TLF-1 uptake and localization. The localization of fluorescently tagged TLF-1, Lysotracker® and DAPI after one hour exposure. The panels represent the following sub-species: *T. b. brucei*, STIB247; *T. b. rhodesiense*, Baganzi; *T. b. gambiense*, STIB386S; *T. b. gambiense*, STIB386R and *T. b. gambiense*, ELIANE.

In order to determine if group 2 *T. b. gambiense* parasites have a functional HpHbR and so take up TLF-1 and traffic the complex to the lysosome in a similar manner to *T. b. brucei*, the uptake of labeled TLF-1 was examined in STIB386S and STIB386R lines in relation to the other parasite strains. TLF-1 was tagged with alexafluor®488 and its uptake analyzed by microscopy in conjunction with the commercial dye Lysotracker®, which labeled acidic vesicles including the lysosome. As previously reported, no labeled TLF-1 was detected in the group 1 *T. b. gambiense* strain parasites indicating a lack of TLF-1 uptake [36]. However the *T. b. brucei* strain STIB247, *T. b. rhodesiense* strain, Baganzi, and both the isogenic group 2 *T. b. gambiense* lines STIB386S and STIB386R lines all showed internalization of TLF-1 within one hour and co-localization of the TLF-1 with acidic vesicles (Figure 4). The mean correlation co-efficients (R) estimating the degree of correlation between the position of TLF-1 and Lysotracker® were: STIB247, R = 0.9 (n = 14); STIB386S, R = 0.68 (n = 18); STIB386R, R = 0.63 (n = 19); Baganzi, R = 0.72 (n = 28). This indicates that the HpHbR protein of group 2 is functional and that reducing uptake of TLF-1 is not a potential resistance mechanism for group 2 *T. b. gambiense* strain STIB386 in contrast to group 1 *T. b. gambiense*, which has been shown to not only have reduced expression of the gene but also a reduced function of the protein due to several amino acid substitutions within the open reading frame (ORF) [36]. Sequence analysis of the ORF of the *HpHbR* gene revealed several differences in sequence (Figure S3). In order to investigate if there was a difference in the rate of uptake of TLF-1 between the different strains, TLF-1 uptake was examined over a four-hour time course and compared to the uptake of bovine HDL. The STIB247, Baganzi and STIB386S lines all took up TLF-1 rapidly whereas STIB386R took up TLF-1 at a slower rate that reached an asymptote at approximately 60% (Figure S4). In contrast, type 1 *T. b. gambiense* showed no visible uptake of TLF-1 during the four-hour exposure.

### Recombinant APOL1 activity, uptake and localization

**Figure 5:**
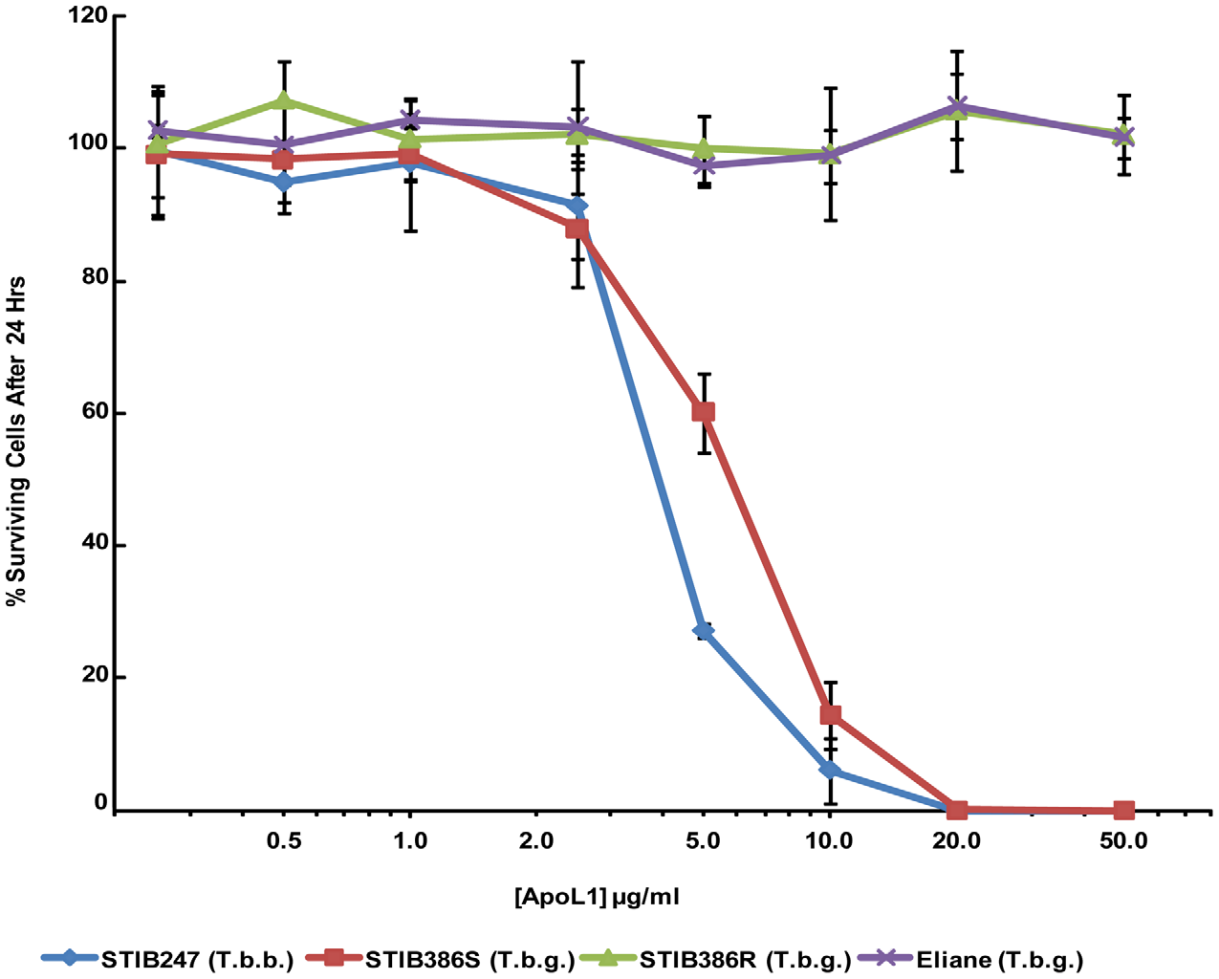
Recombinant APOL1 activity. The percentage of viable motile cells measured after 24-hour exposure to various concentrations of recombinant APOL1 (compared to no APOL1 control) for the *T. b. brucei* strain STIB247, the resistant & sensitive isogenic lines of the group 2 *T. b. gambiense* STIB386 and the group 1 *T. b. gambiense* ELIANE. Standard error is indicated (n = 2).

The knowledge that group 1 *T. b. gambiense* parasites have evolved to avoid TLF-1 uptake raises the possibility that this sub-species group's resistance strategy is based on avoidance and it is inherently susceptible to APOL1 mediated lysis. However, this fact is difficult to reconcile with what is known about TLF-1 and TLF-2 uptake in these parasites. TLF-1 enters trypanosomes not only via the HpHbR but also via a low affinity receptor that is as yet unidentified; therefore a reduction in two TLF-1 receptors is required for protection from TLF-1. In addition, TLF-2 is internalized in a different manner to TLF-1 that does not involve HPR binding [14]. Unfortunately we were not able to work directly with active TLF-2 but it is possible to assay the different strains for susceptibility to APOL1 using recombinant protein. Recombinant APOL1 has been shown in numerous experiments to be toxic to *T. b. brucei* and is internalized via fluid phase endocytosis [21], [61], [62]. Therefore, uptake of this recombinant protein would be unavoidable. The capacity for recombinant APOL1 to lyse group 1 *T. b. gambiense*, in addition to both sensitive and resistant forms of group 2 *T. b. gambiense* and *T. b. brucei*, was examined for several concentrations of protein. Recombinant APOL1 is able to lyse *T. b. brucei* and sensitive group 2 *T. b. gambiense* fully after 24 hours. Lysis occurs at even low concentrations of protein, which approach physiological levels [63]. The resistant group 2 *T. b. gambiense* strain and the group 1 *T. b. gambiense* strain were unaffected by APOL1, even at high concentrations (Figure 5 and Figure S5). This indicates that the difference between sensitive and resistant forms of group 2 *T. b. gambiense* is not due to differences in uptake but a difference in sensitivity to APOL1.

**Figure 6:**
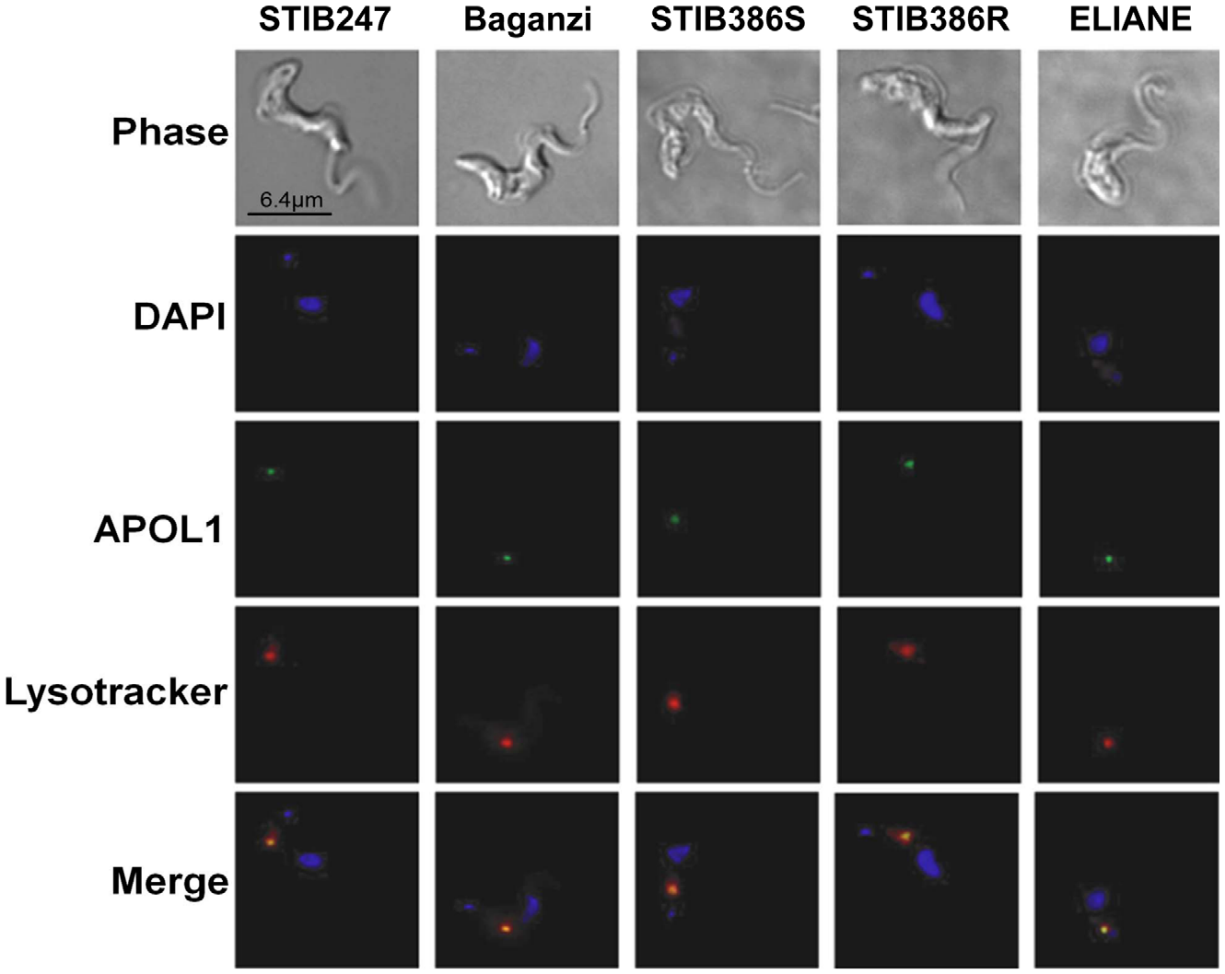
Recombinant APOL1 localization. The localization of Alexa488 labeled anti-pentaHis antibody, Lysotracker® & DAPI after four hour exposure to recombinant ApoL1 featuring a 6×His tag. The panels represent the following sub-species: *T. b. brucei*, STIB247; *T. b. rhodesiense*, Baganzi; *T. b. gambiense*, STIB386S; *T. b. gambiense*, STIB386R and *T. b. gambiense*, ELIANE.

Although in other *T. brucei* lines recombinant APOL1 was internalized and trafficked to the acidic compartments [21], [61], [62], it is possible that the group 1 *T. b. gambiense* and resistant group 2 *T. b. gambiense* are able redirect APOL1 away from these organelles and avoid APOL1 activation. It is also possible that the recombinant protein is not being trafficked to the acidic compartments so would not reach the site of action. To investigate this, the internalization and location of recombinant APOL1 was visualized in several *T. brucei* lines using fluorescence microscopy (Figure 6). All sub-species showed visible uptake of recombinant APOL1 within four hours. The position of highest fluorescence correlates with the lysotracker dye, including the group 1 *T. b. gambiense* strain ELIANE and the resistant group 2 *T. b. gambiense* strain STIB386R. The mean correlation co-efficient (R) of the recombinant APOL1 and Lysotracker® field was estimated for each parasite line: STIB247, R = 0.87 (n = 16); STIB386S, R = 0.81 (n = 13); STIB386R, R = 0.89 (n = 15); Baganzi, R = 0.76 (n = 15); ELIANE, R = 0.85 (n = 18). This would indicate that both group 1 *T. b gambiense* and resistant group 2 *T. b. gambiense* possess the ability to resist the lytic effects of APOL1 in the lysosome. These results are in stark contrast to data suggesting TLF-1 shows reduced uptake in group 1 *T. b. gambiense* cells over short-term due to reduced expression and activity of HpHbR.

In summary, *T. b. gambiense* group 2 parasites appear to express the *HpHbR* gene and have a functional protein that facilitates the uptake of TLF-1, unlike that of *T. b. gambiense* group 1. These results indicate that important differences in the cell biology of human serum resistance exist between the *T. b. gambiense* group 2 parasites and *T. b. gambiense* group 1.

## Discussion

The group 2 *T. b. gambiense* strain STIB386 exhibits a variable phenotype as previously described [31], [64], but continuous exposure to a low concentration of normal human serum maintains the resistant phenotype indefinitely allowing comparisons between isogenic sensitive and resistant forms. Analysis of these parasites indicates that both sensitive and resistant parasites internalize TLF-1. As resistance to lysis by normal human serum and TLF-1 correlates with resistance to recombinant APOL1, it would appear that the resistance mechanism of group 2 *T. b. gambiense* is able to either neutralize or compensate for the effects of the protein. This allows it to overcome both TLF-1 and TLF-2 particles. This is in contrast to group 1 *T. b. gambiense* parasites that avoid uptake of the TLF-1 particle. However the innate resistance of both group 1 and group 2 *T. b. gambiense* to APOL1 might be based on the same mechanism. This resistance is distinct from the *SRA* gene mediated mechanism in *T. b. rhodesiense*, although the mechanism could still involve an inhibitory protein similar to SRA.

Previous attempts to characterize human serum resistance/sensitivity in group 2 *T. b. gambiense* have relied on examination of the TxTat strain [64]. However, some TxTat lines have subsequently been shown to be *T. b. rhodesiense* [65]. Examination of the human serum resistance phenotype in TxTat suggested that a switch between resistant and sensitive forms was always accompanied by a switch in variable antigen type (VAT), indicating that the resistance mechanism is closely related to antigenic variation [49], [64], consistent with these isolates being *T. b. rhodesiense.* Our sequencing data show that identical *ESAG6* and *ESAG7* gene copies are expressed in both strains of STIB386 suggesting that, irrespective of VSG expression, the same dominant ES is being used in both sensitive and resistant populations and that resistance may be unrelated to the use of a particular ES, as it is in *T. b. rhodesiense*. While it is intuitive to assume any variability in expression in trypanosomes is due to expression site switching, expression stochasticity is a common feature to many eukaryotes and can arise from several different mechanisms [66].

As previously reported, the group 1 *T. b. gambiense* strain ELIANE does not appear to internalize and concentrate TLF-1 to any discernable degree over the short term and would appear to employ an avoidance of uptake strategy to prevent lysis by TLF-1. This can be explained by both substantial down-regulation and/or loss of function in the HpHbR involved in internalizing TLF-1 [36]. Alternately, TLF-1 may be differentially trafficked by ELIANE to RAB-11 compartments rather than the lysosome, rapidly removing the toxic particles from the cell and so would not be detected in this assay. There are two group 1 *T. b. gambiense* specific polymorphisms in the 3′ UTR of *HpHbR* that could potentially contribute to mRNA instability and reduced expression of *HpHbR* to affect short-term uptake of TLF-1. There are also several polymorphisms in the ORF of *HpHbR* that appear to affect the function of HpHbR in group 1 *T. b. gambiense* [36]. This has been demonstrated by ectopically expressing the group 1 *T. b. gambiense* version of the gene into an artificially selected L427 HpHbR null mutant, resulting in a failure to restore the full TLF-1 sensitivity phenotype [36].

Interestingly, despite several strains exhibiting a loss of function and expression in the HpHbR receptor [36], group 1 *T. b. gambiense* appear to be fully resistant to the lytic effects of recombinant APOL1. Several hypotheses can be formulated as to why group 1 *T. b. gambiense* avoid TLF-1 despite its inherent resistance to APOL1; these include the hypothesis that the TLF-1 particle evolved before TLF-2 in primates. *T. b. gambiense* may initially have evolved primate infectivity by simply avoiding the TLF-1 particle by modifying receptors for HP/HPR. The sub-species was then unable to use a similar mechanism to avoid uptake of TLF-2 as this particle is not internalized in the same manner [14]. Instead, a second resistance mechanism to counteract APOL1 evolved. Another hypothesis is that the *T. b. gambiense* resistance mechanism is dose dependent and by avoiding TLF-1 uptake is able to avoid the majority of the APOL1 in the human bloodstream. This would suggest that group 1 *T. b. gambiense* may be susceptible to APOL1 lysis if internalized at high concentrations.

The results presented here demonstrate some remarkable features of the variation in human serum resistance mechanisms that have evolved in trypanosome strains. In Eastern Africa, trypanosomes have evolved the SRA protein to neutralize APOL1 and resist lysis by TLF. However, this is not the entire story and some human infective trypanosome strains in these *T. b. rhodesiense* foci do not possess the *SRA* mechanism [23], [67]. The resistance mechanism for these isolates is unknown. While it has been suggested that avoidance of TLF-1 is a resistance strategy in the most prevalent human infective trypanosome, group 1 *T. b. gambiense* [36], this mechanism is not exhibited by the related and sympatric group 2 *T. b. gambiense* suggesting a resistance strategy that is different. It is now clear that human serum resistance in both groups of *T. b. gambiense* involves the ability to either neutralize or resist the effects of APOL1, although resistance is variably expressed in group 2. While it may be that groups 1 and 2 *T. b. gambiense* share a resistance mechanism, population studies suggest that the two groups are not closely related [26], [27], [28], [29], [30], [31], [32], [33], [35], [59], [68], [69], [70]. Taking all of the evidence into account, it would appear that human infectivity has likely evolved in the field on at least four occasions, suggesting there are multiple ways in which these parasites can avoid innate immunity. These facts, coupled with the relative ease with which TLF resistant parasites can be selected from *T. b. brucei* parasites [71] indicates that novel human infective parasites could evolve from the *T. brucei* population. Understanding the processes involved in overcoming these factors will aid in the development of novel intervention strategies.

## Supporting Information

Figure S1 **List of** ***ESAG6*** **and** ***ESAG7*** **variant sequences present in each** ***T. brucei*** **strain determined by unique sequencing reads.**
(DOC)

Figure S2 **Sequence of the expressed** ***ESAG6*** **and** ***ESAG7*** **variable regions for both the isogenic stably sensitive and resistant forms of the group 2** ***T. b. gambiense*** **strain STIB386.** The hypervariable region of each gene is highlighted in red.
(DOC)

Figure S3 **HpHbR ORF and 3′ UTR sequence for several strains of** ***T. brucei*** **.** The open reading frame is denoted by the blue bar. Non-synonymous polymorphisms within the gene are shown in red, synonymous in green. In the 3′UTR polymorphisms are marked solely in red. The two homologues of the HpHbR region in the heterozygous type 2 *T. b. gambiense* strain STIB386 are denoted A and B.
(DOC)

Figure S4 **Uptake of bovine HDL of a comparable size to TLF-1, measured by visible concentrations of AlexaFluor® tagged bovine HDL in the parasite body after 4 hour exposure to labelled bovine HDL.**
(DOC)

Figure S5 **Cell counts of viable motile cells measured after 24-hour exposure to various concentrations of recombinant APOL1 for the** ***T. b. brucei*** **strain STIB247, the resistant and sensitive isogenic lines of the group 2** ***T. b. gambiense*** **STIB386 and the group 1** ***T. b. gambiense*** **ELIANE.** Standard error is indicated (n = 2).
(DOC)

## References

[1] Pays E, Vanhollebeke B (2009). Human innate immunity against African trypanosomes.. Curr Opin Immunol.

[2] WHO (2006). Human African trypanosomiasis (sleeping sickness): epidemiological update.. Wkly Epidemiol Rec.

[3] Pays E, Lips S, Nolan D, Vanhamme L, Pérez-Morga D (2001). The VSG expression sites of *Trypanosoma brucei:* multipurpose tools for the adaptation of the parasite to mammalian hosts.. Mol Biochem Parasitol.

[4] Barry J, McCulloch R (2001). Antigenic variation in trypanosomes: enhanced phenotypic variation in a eukaryotic parasite.. Adv Parasitol.

[5] Thomson R, Molina-Portela P, Mott H, Carrington M, Raper J (2009). Hydrodynamic gene delivery of baboon trypanosome lytic factor eliminates both animal and human-infective African trypanosomes.. Proc Natl Acad Sci U S A.

[6] Seed J, Sechelski J, Loomis M (1990). A survey for a trypanocidal factor in primate sera.. J Protozool.

[7] Poelvoorde P, Vanhamme L, Van Den Abbeele J, Switzer W, Pays E (2004). Distribution of apolipoprotein L-I and trypanosome lytic activity among primate sera.. Mol Biochem Parasitol.

[8] Hajduk SL, Moore DR, Vasudevacharya J, Siqueira H, Torri AF (1989). Lysis of *Trypanosoma brucei* by a toxic subspecies of human high density lipoprotein.. J Biol Chem.

[9] Rifkin MR (1978). Identification of the trypanocidal factor in normal human serum: high density lipoprotein.. Proc Natl Acad Sci U S A.

[10] Raper J, Fung R, Ghiso J, Nussenzweig V, Tomlinson S (1999). Characterization of a novel trypanosome lytic factor from human serum.. Infect Immun.

[11] Widener J, Nielsen MJ, Shiflett A, Moestrup SK, Hajduk S (2007). Hemoglobin is a co-factor of human trypanosome lytic factor.. PLoS Pathog.

[12] Vanhollebeke B, Nielsen MJ, Watanabe Y, Truc P, Vanhamme L (2007). Distinct roles of haptoglobin-related protein and apolipoprotein L-I in trypanolysis by human serum.. Proc Natl Acad Sci U S A.

[13] Vanhollebeke B, De Muylder G, Nielsen M, Pays A, Tebabi P (2008). A haptoglobin-hemoglobin receptor conveys innate immunity to *Trypanosoma brucei* in humans.. Science.

[14] Drain J, Bishop J, Hajduk S (2001). Haptoglobin-related protein mediates trypanosome lytic factor binding to trypanosomes.. J Biol Chem.

[15] Shiflett AM, Bishop JR, Pahwa A, Hajduk SL (2005). Human high density lipoproteins are platforms for the assembly of multi-component innate immune complexes.. J Biol Chem.

[16] Harrington JM, Widener J, Stephens N, Johnson T, Francia M (2010). The plasma membrane of bloodstream-form African trypanosomes confers susceptibility and specificity to killing by hydrophobic peptides.. J Biol Chem.

[17] Hager K, Pierce M, Moore D, Tytler E, Esko J (1994). Endocytosis of a cytotoxic human high density lipoprotein results in disruption of acidic intracellular vesicles and subsequent killing of African trypanosomes.. J Cell Biol.

[18] Molina-Portela Mdel P, Lugli EB, Recio-Pinto E, Raper J (2005). Trypanosome lytic factor, a subclass of high-density lipoprotein, forms cation-selective pores in membranes.. Mol Biochem Parasitol.

[19] Vanhollebeke B, Pays E (2010). The trypanolytic factor of human serum: many ways to enter the parasite, a single way to kill.. Mol Microbiol.

[20] Welburn SC, Picozzi K, Fevre EM, Coleman PG, Odiit M (2001). Identification of human-infective trypanosomes in animal reservoir of sleeping sickness in Uganda by means of serum-resistance-associated (SRA) gene.. Lancet.

[21] Lecordier L, Vanhollebeke B, Poelvoorde P, Tebabi P, Paturiaux-Hanocq F (2009). C-terminal mutants of apolipoprotein L-I efficiently kill both *Trypanosoma brucei brucei* and *Trypanosoma brucei rhodesiense.*. PLoS Pathog.

[22] Lukes J, Raper J (2010). Prophylactic antiparasitic transgenesis for human parasitic disease?. Mol Ther.

[23] De Greef C, Imberechts H, Matthyssens G, Van Meirvenne N, Hamers R (1989). A gene expressed only in serum-resistant variants of *Trypanosoma brucei rhodesiense*.. Mol Biochem Parasitol.

[24] De Greef C, Chimfwembe E, Kihang'a Wabacha J, Bajyana Songa E, Hamers R (1992). Only the serum-resistant bloodstream forms of *Trypanosoma brucei rhodesiense* express the serum resistance associated (SRA) protein.. Ann Soc Belg Med Trop.

[25] Gibson W, Backhouse T, Griffiths A (2002). The human serum resistance associated gene is ubiquitous and conserved in *Trypanosoma brucei rhodesiense* throughout East Africa.. Infect Genet Evol.

[26] Paindavoine P, Pays E, Laurent M, Geltmeyer Y, Le Ray D (1986). The use of DNA hybridization and numerical taxomony in determining relationships between *Trypanosoma brucei* stocks and subspecies.. Parasitology.

[27] Hide G, Cattand P, Le Ray D, Barry JD, Tait A (1990). The identification of *Trypanosoma brucei* subspecies using repetitive DNA sequences.. Molecular and Biochemical Parasitology.

[28] Truc P, Tibayrenc M (1993). Population genetics of *Trypanosoma brucei* in Central Africa: taxonomic and epidemiological significance.. Parasitology.

[29] Jamonneau V, Ravel S, Garcia A, Koffi M, Truc P (2004). Characterization of *Trypanosoma brucei* s.l. infecting asymptomatic sleeping-sickness patients in Cote d'Ivoire: a new genetic group?. Ann Trop Med Parasitol.

[30] Gibson WC, Marshall TF, Godfrey DG (1980). Numerical analysis of enzyme polymorphism: a new approach to the epidemiology and taxonomy of trypanosomes of the subgenus Trypanozoon.. Adv Parasitol.

[31] Mehlitz D, Zillmann U, Scott CM, Godfrey DG (1982). Epidemiological studies on the animal reservoir of Gambiense sleeping sickness. Part III. Characterization of trypanozoon stocks by isoenzymes and sensitivity to human serum.. Tropenmed Parasitol.

[32] Tait A, Babiker EA, Le Ray D (1984). Enzyme variation in *Trypanosoma brucei* spp. I. Evidence for the sub-speciation of *Trypanosoma brucei gambiense*.. Parasitology.

[33] Zillmann U, Mehlitz D, Sachs R (1984). Identity of Trypanozoon stocks isolated from man and a domestic dog in Liberia.. Tropenmed Parasitol.

[34] Gibson WC (1986). Will the real *Trypanosoma b. gambiense* please stand up.. Parasitol Today.

[35] Balmer O, Beadell JS, Gibson W, Caccone A (2011). Phylogeography and Taxonomy of *Trypanosoma brucei.*. PLoS Negl Trop Dis.

[36] Kieft R, Capewell P, Turner CM, Veitch NJ, MacLeod A (2010). Mechanism of *Trypanosoma brucei gambiense* (group 1) resistance to human trypanosome lytic factor.. Proc Natl Acad Sci U S A.

[37] Radwanska M, Claes F, Magez S, Magnus E, Perez-Morga D (2002). Novel primer sequences for polymerase chain reaction-based detection of *Trypanosoma brucei gambiense*.. Am J Trop Med Hyg.

[38] Hirumi H, Hirumi K (1989). Continuous cultivation of *Trypanosoma brucei* blood stream forms in a medium containing a low concentration of serum protein without feeder cell layers.. J Parasitol.

[39] Shiflett AM, Bishop JR, Pahwa A, Hajduk SL (2005). Human high density lipoproteins are platforms for the assembly of multi-component innate immune complexes.. J Biol Chem.

[40] Livak KJ, Schmittgen TD (2001). Analysis of relative gene expression data using real-time quantitative PCR and the 2(-Delta Delta C(T)) Method.. Methods.

[41] Rozen S, Skaletsky H (2000). Primer3 on the WWW for general users and for biologist programmers.. Methods Mol Biol.

[42] Berberof M, Perez-Morga D, Pays E (2001). A receptor-like flagellar pocket glycoprotein specific to *Trypanosoma brucei gambiense*.. Mol Biochem Parasitol.

[43] Morrison LJ, Tait A, McCormack G, Sweeney L, Black A (2008). *Trypanosoma brucei gambiense* Type 1 populations from human patients are clonal and display geographical genetic differentiation.. Infect Genet Evol.

[44] Becker M, Aitcheson N, Byles E, Wickstead B, Louis E (2004). Isolation of the repertoire of VSG expression site containing telomeres of *Trypanosoma brucei* 427 using transformation-associated recombination in yeast.. Genome Res.

[45] Steverding D, Stierhof Y, Chaudhri M, Ligtenberg M, Schell D (1994). ESAG 6 and 7 products of *Trypanosoma brucei* form a transferrin binding protein complex.. Eur J Cell Biol.

[46] Turner CM, McLellan S, Lindergard LA, Bisoni L, Tait A (2004). Human infectivity trait in *Trypanosoma brucei*: stability, heritability and relationship to sra expression.. Parasitology.

[47] Cooper A, Tait A, Sweeney L, Tweedie A, Morrison L (2008). Genetic analysis of the human infective trypanosome *Trypanosoma brucei gambiense:* chromosomal segregation, crossing over, and the construction of a genetic map.. Genome Biol.

[48] De Greef C, Hamers R (1994). The serum resistance-associated (SRA) gene of *Trypanosoma brucei rhodesiense* encodes a variant surface glycoprotein-like protein.. Mol Biochem Parasitol.

[49] Xong HV, Vanhamme L, Chamekh M, Chimfwembe CE, Van Den Abbeele J (1998). A VSG expression site-associated gene confers resistance to human serum in *Trypanosoma rhodesiense*.. Cell.

[50] Ligtenberg M, Bitter W, Kieft R, Steverding D, Janssen H (1994). Reconstitution of a surface transferrin binding complex in insect form *Trypanosoma brucei.*. EMBO J.

[51] Salmon D, Geuskens M, Hanocq F, Hanocq-Quertier J, Nolan D (1994). A novel heterodimeric transferrin receptor encoded by a pair of VSG expression site-associated genes in *T. brucei.*. Cell.

[52] Steverding D, Stierhof YD, Fuchs H, Tauber R, Overath P (1995). Transferrin-binding protein complex is the receptor for transferrin uptake in *Trypanosoma brucei*.. J Cell Biol.

[53] Young R, Taylor JE, Kurioka A, Becker M, Louis EJ (2008). Isolation and analysis of the genetic diversity of repertoires of VSG expression site containing telomeres from *Trypanosoma brucei gambiense*, *T. b. brucei* and *T. equiperdum*.. BMC Genomics.

[54] Berriman M, Ghedin E, Hertz-Fowler C, Blandin G, Renauld H (2005). The genome of the African trypanosome *Trypanosoma brucei*.. Science.

[55] Jackson AP, Sanders M, Berry A, McQuillan J, Aslett MA (2010). The genome sequence of *Trypanosoma brucei gambiense*, causative agent of chronic human african trypanosomiasis.. PLoS Negl Trop Dis.

[56] Becker M, Aitcheson N, Byles E, Wickstead B, Louis E (2004). Isolation of the repertoire of VSG expression site containing telomeres of *Trypanosoma brucei* 427 using transformation-associated recombination in yeast.. Genome Res.

[57] Bringaud F, Biteau N, Donelson JE, Baltz T (2001). Conservation of metacyclic variant surface glycoprotein expression sites among different trypanosome isolates.. Mol Biochem Parasitol.

[58] Jackson AP, Sanders M, Berry A, McQuillan J, Aslett MA (2010). The genome sequence of *Trypanosoma brucei gambiense*, causative agent of chronic human african trypanosomiasis.. PLoS Neglected Tropical Diseases.

[59] Gibson WC (1986). Will the real *Trypanosoma b. gambiense* please stand up.. Parasitology Today.

[60] Hide G, Welburn SC, Tait A, Maudlin I (1994). Epidemiological relationships of *Trypanosoma brucei* stocks from South East Uganda: evidence for different population structures in human infective and non-human infective isolates.. Parasitology.

[61] Molina-Portela MP, Samanovic M, Raper J (2008). Distinct roles of apolipoprotein components within the trypanosome lytic factor complex revealed in a novel transgenic mouse model.. J Exp Med.

[62] Vanhamme L, Paturiaux-Hanocq F, Poelvoorde P, Nolan DP, Lins L (2003). Apolipoprotein L-I is the trypanosome lytic factor of human serum.. Nature.

[63] Page NM, Olano-Martin E, Lanaway C, Turner R, Minihane AM (2006). Polymorphisms in the Apolipoprotein L1 gene and their effects on blood lipid and glucose levels in middle age males.. Genes Nutr.

[64] Ortiz JC, Sechelski JB, Seed JR (1994). Characterization of human serum-resistant and serum-sensitive clones from a single *Trypanosoma brucei gambiense* parental clone.. J Parasitol.

[65] Grab DJ, Kennedy PG (2008). Traversal of human and animal trypanosomes across the blood-brain barrier.. J Neurovirol.

[66] Kaern M, Elston TC, Blake WJ, Collins JJ (2005). Stochasticity in gene expression: from theories to phenotypes.. Nat Rev Genet.

[67] Enyaru JC, Matovu E, Nerima B, Akol M, Sebikali C (2006). Detection of *T.b. rhodesiense* trypanosomes in humans and domestic animals in south east Uganda by amplification of serum resistance-associated gene.. Ann N Y Acad Sci.

[68] Godfrey DG, Kilgour V (1976). Enzyme electrophoresis in characterizing the causative organism of Gambian trypanosomiasis.. Trans R Soc Trop Med Hyg.

[69] Mathieu-daude F, Tibayrenc M (1994). Isozyme Variability of *Trypanosoma-brucei* S-L - Genetic, Taxonomic, and Epidemiologic Significance.. Exp Parasitol.

[70] Stevens JR, Tibayrenc M (1996). *Trypanosoma brucei* sl: Evolution, linkage and the clonality debate.. Parasitology.

[71] Faulkner SD, Oli MW, Kieft R, Cotlin L, Widener J (2006). In vitro generation of human high-density-lipoprotein-resistant *Trypanosoma brucei brucei*.. Eukaryot Cell.

