# Supplementary material for: Differences between Trypanosoma brucei gambiense Groups 1 and 2 in Their Resistance to Killing by Trypanolytic Factor 1
Source: PLoS Negl Trop Dis. 2011 Sep 6;5(9):e1287. doi: 10.1371/journal.pntd.0001287 (PMC3167774; doi:10.1371/journal.pntd.0001287)
Supplement: Figure S1 — List of ESAG6 and ESAG7 variant sequences present in each T. brucei strain determined by unique sequencing reads. (DOC) [file pntd.0001287.s001.doc]

**Figure S1**

*ESAG6* variants

| **STIB386** | **TREU927** | **STIB247** | **DAL927** |
| --- | --- | --- | --- |
| Tb427.BES65.2 | Tb427.BES65.2 | 13J3.10 | Tb427.BES65.2 |
| Tb427.BES65.1 | Tb427.BES65.1 | N19B2.165 | Tb427.BES65.1 |
| Tb427.BES122.2 | Tb427.BES122.2 | H25N7.15 | Tb427.BES122.2 |
| Tb427.BES4.2 | Tb427.BES4.2 | Tb427.BES65.2 | Tb427.BES126.3 |
| Tb427.BES5.2 | Tb427.BES5.2 | Tb427.BES65.1 | Tb427.BES153.2 |
| Tb427.BES28.4 | Tb427.BES28.4 | Tb427.BES122.2 | Tb427.BES153.3 |
| Tb427.BES126.3 | Tb427.BES126.3 | Tb427.BES4.2 | Tb427.BES29.2 |
| N19B2.165 | N19B2.165 | Tb427.BES5.2 | Tb427.BES56.2 |
| Tb427.BES129.4 | Tb427.BES129.4 | Tb427.BES28.4 |  |
| Tb427.BES153.2 | Tb427.BES153.2 | Tb427.BES126.3 |  |
| Tb427.BES153.3 | Tb427.BES153.3 | N19B2.165 |  |
| Tb427.BES40.3 | Tb427.BES40.3 | Tb427.BES153.2 |  |
| Tb427.BES134.3 | Tb427.BES134.3 | Tb427.BES153.3 |  |
| 13J3.10 | 13J3.10 | Tb427.BES134.3 |  |
| H25N7.15 | H25N7.15 | Tb427.BES98.2 |  |
| Tb427.BES98.2 | Tb427.BES98.2 | Tb427.BES29.2 |  |
| Tb427.BES29.2 | Tb427.BES29.2 | Tb427.BES56.2 |  |
| Tb427.BES56.2 | Tb427.BES56.2 | Tb427.BES59.3 |  |
| Tb427.BES59.3 | Tb427.BES59.3 | Tb427.BES15.3 |  |
| Tb427.BES15.3 | Tb427.BES15.3 | Tb427.BES51.3 |  |
| Tb427.BES51.3 | Tb427.BES51.3 | Tb427.BES10.2 |  |
| Tb427.BES10.2 | Tb427.BES10.2 |  |  |

*ESAG7* variants

| **STIB386** | **TREU927** | **STIB247** | **DAL972** |
| --- | --- | --- | --- |
| Tb427.BES122.1 | Tb427.BES122.1 | Tb427.BES122.1 | Tb427.BES28.2 |
| Tb427.BES4.1 | Tb427.BES4.1 | Tb427.BES4.1 | Tb427.BES28.1 |
| Tb427.BES28.2 | Tb427.BES28.2 | Tb427.BES28.2 | Tb427.BES28.3 |
| Tb427.BES28.1 | Tb427.BES28.1 | Tb427.BES28.1 | Tb427.BES126.2 |
| Tb427.BES28.3 | Tb427.BES28.3 | Tb427.BES28.3 | Tb427.BES134.2 |
| Tb427.BES126.2 | Tb427.BES126.2 | Tb427.BES126.2 | Tb427.BES64.1 |
| Tb427.BES5.1 | Tb427.BES5.1 | Tb427.BES5.1 | Tb427.BES29.3 |
| N19B2.155 | N19B2.155 | N19B2.155 | Tb427.BES56.3 |
| Tb427.BES129.2 | Tb427.BES129.2 | Tb427.BES129.2 | Tb427.BES10.1 |
| N19B2.160 | Tb427.BES129.3 | Tb427.BES40.2 |  |
| Tb427.BES40.2 | Tb427.BES40.2 | Tb427.BES134.2 |  |
| Tb427.BES134.2 | Tb427.BES134.2 | 13J3.09 |  |
| 13J3.09 | 13J3.09 | H25N7.14 |  |
| Tb427.BES64.1 | H25N7.14 | Tb427.BES64.1 |  |
| Tb427.BES98.1 | Tb427.BES64.1 | Tb427.BES98.1 |  |
| Tb427.BES29.3 | Tb427.BES98.1 | Tb427.BES29.3 |  |
| Tb427.BES56.3 | Tb427.BES29.3 | Tb427.BES56.3 |  |
| Tb427.BES59.2 | Tb427.BES56.3 | Tb427.BES59.2 |  |
| Tb427.BES15.2 | Tb427.BES59.2 | Tb427.BES15.2 |  |
| Tb427.BES51.2 | Tb427.BES15.2 | Tb427.BES51.2 |  |
| Tb427.BES10.1 | Tb427.BES51.2 | Tb427.BES10.1 |  |
|  | Tb427.BES10.1 |  |  |
