# Supplementary material for: Differences between Trypanosoma brucei gambiense Groups 1 and 2 in Their Resistance to Killing by Trypanolytic Factor 1
Source: PLoS Negl Trop Dis. 2011 Sep 6;5(9):e1287. doi: 10.1371/journal.pntd.0001287 (PMC3167774; doi:10.1371/journal.pntd.0001287)
Supplement: Figure S2 — Sequence of the expressed ESAG6 and ESAG7 variable regions for both the isogenic stably sensitive and resistant forms of the group 2 T. b. gambiense strain STIB386. The hypervariable region of each gene is highlighted in red. (DOC) [file pntd.0001287.s002.doc]

**Figure S2.**

386R_ESAG6(BES126) CATTCCAGCAGGAGTTGGAGGAAATGAGGAACGCATCCGCGTTAGCAGCAGCTGCAGCTG

386S_ESAG6(BES126) CATTCCAGCAGGAGTTGGAGGAAATGAGGAACGCATCCGCGTTAGCAGCAGCTGCAGCTG

386R_ESAG6(BES126) GGATAGCAGCTGGAAGACTGGAAGAGTGGATTTTTGTATTTGCACAGGCAGCAGGCAGGT

386S_ESAG6(BES126) GGATAGCAGCTGGAAGACTGGAAGAGTGGATTTTTGTATTTGCACAGGCAGCAGGCAGGT

Hyper variable region – identifies to L427 ES 126

386R_ESAG6(BES126) CATCACAGTTTTGCATAAGCACGGGGAAGACTGGGCCAGCAGAATACAACAACTTGCAAG

386S_ESAG6(BES126) CATCACAGTTTTGCATAAGCACGGGGAAGACTGGGCCAGCAGAATACAACAACTTGCAAG

386R_ESAG6(BES126) AATGTTTTGATGGAACAATTGGACCTGAAACCCTTTACAAAATTGAGGATTCACGTGTAA

386S_ESAG6(BES126) AATGTTTTGATGGAACAATTGGACCTGAAACCCTTTACAAAATTGAGGATTCACGTGTAA

386R_ESAG6(BES126) AAGAGTCGGCGAAGACAAGATTGCTACTCCATGAAGTGTTATTATCCATTTCCTTCGGTA

386S_ESAG6(BES126) AAGAGTCGGCGAAGACAAGATTGCTACTCCATGAAGTGTTATTATCCATTTCCTTCGGTA

386R_ESAG6(BES126) GCTTGGGTGCGGAAAATATCCGAGGAGGAAATGGGAAGGATGGATGTAATTTGGTGAGAA

386S_ESAG6(BES126) GCTTGGGTGCGGAAAATATCCGAGGAGGAAATGGGAAGGATGGATGTAATTTGGTGAGAA

386R_ESAG6(BES126) CAGACAATAACGGTATATTAAAGGGGGGGTCACCGACACGGCACAACTTAACGTGGGGTG

386S_ESAG6(BES126) CAGACAATAACGGTATATTAAAGGGGGGGTCACCGACACGGCACAACTTAACGTGGGGTG

386R_ESAG6(BES126) GTGGAGTGATGAATTTTGGATCCTATCAAAACGGAAGTATGTACGTTGAGGGTGGTGAAT

386S_ESAG6(BES126) GTGGAGTGATGAATTTTGGATCCTATCAAAACGGAAGTATGTACGTTGAGGGTGGTGAAT

386R_ESAG6(BES126) ACGGTGATGCTACAGAGTACGGTGCAGTACGGTGGACCGAAGATCCTAGTAAAGTGAGCA

386S_ESAG6(BES126) ACGGTGATGCTACAGAGTACGGTGCAGTACGGTGGACCGAAGATCCTAGTAAAGTGAGCA

386R_ESAG6(BES126) TATTTAAGGATGTCATTCGCCTGTTTGCGCGGTTCCAAGAAGCAAAAAATGCAGTGATGA

386S_ESAG6(BES126) TATTTAAGGATGTCATTCGCCTGTTTGCGCGGTTCCAAGAAGCAAAAAATGCAGTGATGA

386R_ESAG6(BES126) AGAAAATAAAAACTACTGTGGATGAATTGACGAAATGTATAGGGCAGAAGGAGGCTGAAC

386S_ESAG6(BES126) AGAAAATAAAAACTACTGTGGATGAATTGACGAAATGTATAGGGCAGAAGGAGGCTGAAC

386R_ESAG6(BES126) TCACTAATGATCAGATTTACGAGGAATTTATCTGGGAGACCATAAACAGATTGGAGCTGT

386S_ESAG6(BES126) TCACTAATGATCAGATTTACGAGGAATTTATCTGGGAGACCATAAACAGATTGGAGCTGT

386R_ESAG6(BES126) CAAAGAGAGTGAGTGAACAA

386S_ESAG6(BES126) CAAAGAGAGTGAGTGAACAA

386R_ESAG7(BES126) CGAGCGGCCGCCAGTGTGATGGATATCTGCAGAATTCGCCCTTCCGGAATTCGCTATTAT

386S_ESAG7(BES126) CGAGCGGCCGCCAGTGTGATGGATATCTGCAGAATTCGCCCTTCCGGAATTCGCTATTAT

386R_ESAG7(BES126) TAGAACAGTTTCTGTACTATATTGTAACGAAGCGGAAAGTTTTAAAAGCATAGTTTAAAA

386S_ESAG7(BES126) TAGAACAGTTTCTGTACTATATTGTAACGAAGCGGAAAGTTTTAAAAGCATAGTTTAAAA

386R_ESAG7(BES126) AAGTGATAAGGATGAGATTTTGGTTTGTGTTGTTGGCCCTTTTGGGAAAAGAAACATATG

386S_ESAG7(BES126) AAGTGATAAGGATGAGATTTTGGTTTGTGTTGTTGGCCCTTTTGGGAAAAGAAACATATG

386R_ESAG7(BES126) CGTATGAAAATGAAAGGAATGCATTAAACGCAACCGCCGCTAATAAAGTGTGTGCGCTAT

386S_ESAG7(BES126) CGTATGAAAATGAAAGGAATGCATTAAACGCAACCGCCGCTAATAAAGTGTGTGCGCTAT

386R_ESAG7(BES126) CGACCTATCTTAAAGGAATAGCGCACAGAGTAAACAGCGAAAGTGCTGTGGTTACGGAAA

386S_ESAG7(BES126) CGACCTATCTTAAAGGAATAGCGCACAGAGTAAACAGCGAAAGTGCTGTGGTTACGGAAA

386R_ESAG7(BES126) AACTATCAGATTTGAAAATGAGAAGCATCCAGTTGCAGCTAACAGTAATGCGAAACAGAG

386S_ESAG7(BES126) AACTATCAGATTTGAAAATGAGAAGCATCCAGTTGCAGCTAACAGTAATGCGAAACAGAG

386R_ESAG7(BES126) ATCCTTCTGGCGAGAAGGATTGTAAAGACATCAGGACACTCTTGAAAACAGTATTGAGGA

386S_ESAG7(BES126) ATCCTTCTGGCGAGAAGGATTGTAAAGACATCAGGACACTCTTGAAAACAGTATTGAGGA

386R_ESAG7(BES126) ATGAGTTTACATTCCAGCAGGAGTTGGAGGAAATGAGGAACGCATCCGCGTTAGCAGCAG

386S_ESAG7(BES126) ATGAGTTTACATTCCAGCAGGAGTTGGAGGAAATGAGGAACGCATCCGCGTTAGCAGCAG

386R_ESAG7(BES126) CTGCAGCTGGGATAGCAGCCGGAAGACTGGAAGAATGGATTTTTGTATTTGCACAGGCAG

386S_ESAG7(BES126) CTGCAGCTGGGATAGCAGCCGGAAGACTGGAAGAATGGATTTTTGTATTTGCACAGGCAG

386R_ESAG7(BES126) CAGGCAGGTCATCACAGTTTTGCATAAGCGTGGGGAAGCATATCCCGGCTGAGCACGGCA

386S_ESAG7(BES126) CAGGCAGGTCATCACAGTTTTGCATAAGCGTGGGGAAGCATATCCCGGCTGAGCACGGCA

386R_ESAG7(BES126) ACTTGCAAGAATGTTTTGACGGAACAATTGGACCTGAAACCCTTTACAAAATTGAGGATT

386S_ESAG7(BES126) ACTTGCAAGAATGTTTTGACGGAACAATTGGACCTGAAACCCTTTACAAAATTGAGGATT

386R_ESAG7(BES126) CACGTGTAAAAGAGTCGGCAAAGACAAGCTTGCAACTCCATGAAGTGTTATCATCCATTT

386S_ESAG7(BES126) CACGTGTAAAAGAGTCGGCAAAGACAAGCTTGCAACTCCATGAAGTGTTATCATCCATTT

386R_ESAG7(BES126) CCTTCAATAGCTTGGGTGCGGAAAGTATTGTTGAGCAAGGAGAAAACAGAGGATGTAACC

386S_ESAG7(BES126) CCTTCAATAGCTTGGGTGCGGAAAGTATTGTTGAGCAAGGAGAAAACAGAGGATGTAACC

386R_ESAG7(BES126) TAATGCGGACCGCGTACGGAGGTTTGCTGGAAAGGTGTTTGTTTGAATCGCAACTTCACA

386S_ESAG7(BES126) TAATGCGGACCGCGTACGGAGGTTTGCTGGAAAGGTGTTTGTTTGAATCGCAACTTCACA

386R_ESAG7(BES126) TGGGGTGCCGGAGTGTTGAATTTCGGATACTGTGTGGCGGGGAACCTAAAAATAAAAGGG

386S_ESAG7(BES126) TGGGGTGCCGGAGTGTTGAATTTCGGATACTGTGTGGCGGGGAACCTAAAAATAAAAGGG

Hyper variable region – identifies to L427 ES 126

386R_ESAG7(BES126) GGAGAATACGGTGATGTCAGTTCCCACGATGCGGTACGGTGGACCGAAGATCCTAGTAAA

386S_ESAG7(BES126) GGAGAATACGGTGATGTCAGTTCCCACGATGCGGTACGGTGGACCGAAGATCCTAGTAAA

386R_ESAG7(BES126) GTGAGCATATTTAAAGGATGTCATTCGCCTGTTTGCGCGGTTCAAAGAAGCAAAAAATGC

386S_ESAG7(BES126) GTGAGCATATTTAAAGGATGTCATTCGCCTGTTTGCGCGGTTCAAAGAAGCAAAAAATGC

386R_ESAG7(BES126) AGTGATGTCTAGAGCAAGGGCGAATTCCAGCACACTGGCGGCCGTTACTA

386S_ESAG7(BES126) AGTGATGTCTAGAGCAAGGGCGAATTCCAGCACACTGGCGGCCGTTACTA
