# Supplementary material for: Differences between Trypanosoma brucei gambiense Groups 1 and 2 in Their Resistance to Killing by Trypanolytic Factor 1
Source: PLoS Negl Trop Dis. 2011 Sep 6;5(9):e1287. doi: 10.1371/journal.pntd.0001287 (PMC3167774; doi:10.1371/journal.pntd.0001287)
Supplement: Figure S3 — HpHbR ORF and 3′ UTR sequence for several strains of T. brucei . The open reading frame is denoted by the blue bar. Non-synonymous polymorphisms within the gene are shown in red, synonymous in green. In the 3′UTR polymorphisms are marked solely in red. The two homologues of the HpHbR region in the heterozygous type 2 T. b. gambiense strain STIB386 are denoted A and B. (DOC) [file pntd.0001287.s003.doc]

**Figure S3.**

|Gene-> _

TREU927 (T.b.b) ATGGAGAAACCGTCTTGCAGGGGTGCCGGTTGGGCGCAGCTTTTGTGGTGTTACGGCACC

Eliane (Gp 1 T.b.g) ATGGAGAAACCGTCTTGCAGGGGTGCCGGTTGGGCGCAGCTTTTGTGGTGTTACGGCACC

STIB386A (Gp 2 T.b.g) ATGGAGAAACCGTCTTGCAGGGGTGCCGGTTGGGCGCAGCTTTTGTGGTGTTACGGCACC

STIB386B (Gp 2 T.b.g) ATGGAGAAACCGTCTTGCAGGGGTGCTGGTTGGGCGCAGCTTTTGTGGTGTTACGGCACC

_

TREU927 (T.b.b) TGTTGCGCTCTACTCCTCCGCCTGATAGTTGAAGCCAGTCAAGCTGCTGAGGGTTTAAAA

Eliane (Gp 1 T.b.g) TGTTGCGCTCTACTCCTCCGCCTGATAGTTGAAGCCAGTCAAGCTGCTGAGGGTTTAAAA

STIB386A (Gp 2 T.b.g) TGTTGCGCTCTACTCCTCCGCCTGATAGTTGAAGCCAGTCAAGCTGCTGAGGGTTTAAAA

STIB386B (Gp 2 T.b.g) TGTTGCGCTCTACTCCTCCGCCTGATAGTTGAAGCCAGTCAAGCTGCTGAGGGTTTAAAA

_

TREU927 (T.b.b) ACCAAAGACGAAGTTGAGAAGGCGTGCCATCTTGCACAACAACTCAAAGAAGTTTCAATT

Eliane (Gp 1 T.b.g) ACCAAAGACGAAGTTGAGAAGGCGTGCCATCTTGCACAACAACTCAAAGAAGTTTCAATT

STIB386A (Gp 2 T.b.g) ACCAAAGACGAAGTTGAGAAGGCGTGCCATCTTGCACAACAACTCAAAGAAGTTTCAATT

STIB386B (Gp 2 T.b.g) ACCAAAGACGAAGTTGAGAAGGCGTGCCATCTTGCACAACAACTCAAAGAAGTTTCAATT

_

TREU927 (T.b.b) ACTTTGGGAGTTATTTACCGGACCACTGAACGACACTCCGTGCAAGTTGAAGCGCATAAA

Eliane (Gp 1 T.b.g) ACTTTGGGAGTTATTTACCGGACCACTGAACGACACTCCGTGCAAGTTGAAGCGCATAAA

STIB386A (Gp 2 T.b.g) ACTTTGGGAGTTATTTACCGGACCACTGAACGACACTCCGTGCAAGTTGAAGCGCATAAA

STIB386B (Gp 2 T.b.g) ACTTTGGGAGTTATTTACCGGACCACTGAACGACACTCCGTGCAAGTTGAAGCGCATAAA

_

TREU927 (T.b.b) ACAGCCATTGACAAACATGCGGATGCGGTGTCGCGAGCTGTGGAGGCGCTCACGAGGGTG

Eliane (Gp 1 T.b.g) ACAGCCATTGACAAACATGCGGATGCGGTGTCGCGAGCTGTGGAGGCGCTCACGAGGGTG

STIB386A (Gp 2 T.b.g) ACAGCCATTGACAAACATGCCGATGCGGTGTCGCGAGCTGTGGAGGCGCTCACGAGGGTG

STIB386B (Gp 2 T.b.g) ACAGCCATTGACAAACATGCGGATGCGGTGTCGCGAGCTGTGGAGGCGCTCACGAGGGTG

_

TREU927 (T.b.b) GATGTGGCACTTCAGCGATTGAAAGAACTCGGGAAGGCGAATGACACGAAGGCGGTGAAA

Eliane (Gp 1 T.b.g) GATGTGGCACTTCAGCGATTGAAAGAACTCGGGAAGGCCAATGACACGAAGGCGGTGAAA

STIB386A (Gp 2 T.b.g) GATGTGGCACTTCAGCGATTGAAAGAACTCGGGAAGGCGAATGACACGAAGGCGGTGAAA

STIB386B (Gp 2 T.b.g) GATGTGGCACTTCAGCGATTGAAAGAACTCGGGAAGGCGAATGACACGAAGGCGGTGAAA

_

TREU927 (T.b.b) ATTATCGAGAACATTACCTCCGCCAGAGAAAATCTCGCTCTCTTCAATAACGAAACGCAG

Eliane (Gp 1 T.b.g) ATTATCGAGAACATTACCTCCGCCAGAGAAAATCTCGCTCTCTTCAATAACGAAACGCAG

STIB386A (Gp 2 T.b.g) ATTATCGAGAACATTACCTCCGCCAGAGAAAATCTCGCTCTCTTCAATAACGAAACGCAG

STIB386B (Gp 2 T.b.g) ATTATCGAGAACATTACCTCCGCCAGAGAAAATCTCGCTCTCTTCAATAACGAAACGCAG

_

TREU927 (T.b.b) GCCGTACTGACGGCGAGGGATCATGTGCATAAGCATAGGGCCGCGGCATTGCAGGGGTGG

Eliane (Gp 1 T.b.g) GCCGTACTGACGGCGAGGGATCATGTGCATAAGCATAGGGCCGCGGCATTGCAGGGGTGG

STIB386A (Gp 2 T.b.g) GCCGTACTGACGGCGAGGGATCATGTGCATAAGCATAGGGCCGCGGCATTGCAGGGGTGG

STIB386B (Gp 2 T.b.g) GCCGTACTGACGGCGAGGGATCATGTGCATAAGCATAGGGCCGCGGCATTGCAGGGGTGG

_

TREU927 (T.b.b) TCTGATGCAAAAGAAAAAGGCGATGCCGCCGCAGAGGATGTTTGGGTTCTGCTTAATGCC

Eliane (Gp 1 T.b.g) TCTGATGCAAAAGAAAAAGGCGATGCCGCCGCAGAGGATGTTTGGGTTCTGCTTAATGCC

STIB386A (Gp 2 T.b.g) TCTGATGCAAAAGAAAAAGGCGATGCCGCCGCAGAGGATGTTTGGGTTCTGCTTAATGCC

STIB386B (Gp 2 T.b.g) TCTGATGCAAAAGAAAAAGGCGATGCCGCCGCAGAGGATGTTTGGGTTCTGCTTAATGCC

_

TREU927 (T.b.b) GCAAAAAAAGGTAATGGCAGTGCAGACGTCAAAGCAGCTGCAGAGAAATGCTCGAGATAT

Eliane (Gp 1 T.b.g) GCAAAAAAAGGTAATGGCAGTGCAGACGTCAAGGCAGCTGCAGAGAAATGCTCGAGATAT

STIB386A (Gp 2 T.b.g) GCAAAAAAAGGTAATGGCAGTGCAGACGTCAAGGCAGCTGCAGAGAAATGCTCGAGATAT

STIB386B (Gp 2 T.b.g) GCAAAAAAAGGTAATGGCAGTGCAGACGTCAAGGCAGCTGCAGAGAAATGCTCGAGATAT

_

TREU927 (T.b.b) TCCTCAAGCAGTACTTCAGAAACTGAGTTGCAGAAAGCTATTGACGCCGCCGCTAACGTG

Eliane (Gp 1 T.b.g) TCCTCAAGCAGTACTTCAGAAACTGAGTCGCAGAAAGCTATTGACGCCGCCGCTAACGTG

STIB386A (Gp 2 T.b.g) TCCTCAAGCAGTACTTCAGAAACTGAGTTGCAGAAAGCTATTGACGCCGCCGCTAACGTG

STIB386B (Gp 2 T.b.g) TCCTCAAGCAGTACTTCAGAAACTGAGTTGCAGAAAGCTATTGACGCCGCCGCTAACGTG

_

TREU927 (T.b.b) GGGGGTTTGTCGGCACACAAGTCGAAATATGGCGATGTGCTGAACAAGTTTAAATTGTCT

Eliane (Gp 1 T.b.g) GGGGGTTTGTCGGCACACAAGTCGAAATATGGCGATGTGCTGAACAAGTTTAAATTGTCT

STIB386A (Gp 2 T.b.g) GGGGGTTTGTCGGCACACAAGTCGAAATATGGCGATGTGCTGAACAAGTTTAAATTGTCT

STIB386B (Gp 2 T.b.g) GGGGGTTTGTCGGCACACAAGTCGAAATATGGCGATGTGCTGAACAAGTTTAAATTGTCT

_

TREU927 (T.b.b) AATGCTTCAGTGGGAGCAGTGAGAGACACATCCGGCCGGGGCGGTAAGCATATGGAAAAG

Eliane (Gp 1 T.b.g) AATGCTTCAGTGGGAGCAGTGAGAGACACATCCGGCCGGGGCGGTAAGCATATGGAAAAG

STIB386A (Gp 2 T.b.g) AATGCTTCAGTGGGAGCAGTGAGAGACACATCCGGCCGGGGCGGTAAGCATATGGAAAAG

STIB386B (Gp 2 T.b.g) AATGCTTCAGTGGGAGCAGTGAGAGACACATCCGGCCGGGGCGGTAAGCATATGGAAAAG

_

TREU927 (T.b.b) GTCAATAATGTGGCAAAACTTCTTAAGGATGCAGAGGTTTCTCTTGCAGCTGCAGCAGCC

Eliane (Gp 1 T.b.g) GTCAATAATGTGGCAAAACTTCTTAAGGATGCAGAGGTTTCTCTTGCAGCTGCAGCAGCC

STIB386A (Gp 2 T.b.g) GTCAATAATGTGGCAAAACTTCTTAAGGATGCAGAGGTTTCTCTTGCAGCTGCAGCAGCC

STIB386B (Gp 2 T.b.g) GTCAATAATGTGGCAAAACTTCTTAAGGATGCAGAGGTTTCTCTTGCAGCTGCAGCAGCC

_

TREU927 (T.b.b) GAAATTGAGGAGGTTAAAAATGCACATGAAACAAAAGCACAGGAAGAGATGAAGCGCAAT

Eliane (Gp 1 T.b.g) GAAATTGAGGAGGTTAAAAATGCACATGAAACAAAAGTACAGGAAGAGATGAAGCGCAAT

STIB386A (Gp 2 T.b.g) GAAATTGAGGAGGTTAAAAATGCACATGAAACAAAAGTACAGGAAGAGATGAAGCGCAAT

STIB386B (Gp 2 T.b.g) GAAATTGAGGAGGTTAAAAATGCACATGAAACAAAAGCACAGGAAGAGATGAAGCGCAAT

_

TREU927 (T.b.b) GGGAACCCGATCGAAAATGAATCAGAGACTAATTCAGGGGGGAATGCGGAATCACAAGGT

Eliane (Gp 1 T.b.g) GGGAACCCGATCGAAAATGAATCAGAGACTAATTCAGGGGGGAATGCGGAATCACAAGGT

STIB386A (Gp 2 T.b.g) GGGAACCCGATCGAAAATGAATCAGAGACTAATTCAGGGGGGAATGCGGAATCACAAGGT

STIB386B (Gp 2 T.b.g) GGGAACCCGATCGAAAATGAATCAGGGACTAATTCAGGGGGGAATGCGGAATCACAAGGT

_

TREU927 (T.b.b) AATGGAGATCGTGAAGATAAGAACGACGAGCAACAACAGGTCGATGAGGAGGAAACAAAG

Eliane (Gp 1 T.b.g) AATGGAGATCGTGAAGATAAGAACGACGAGCAACAACAGGTCGATGAGGAGGAAACAAAG

STIB386A (Gp 2 T.b.g) AATGGAGATTGTGAAGATAAGAACGACGAGCAACAACAGGTCGATGAGGAGGAAACAAAG

STIB386B (Gp 2 T.b.g) ACTGGAGATCGTGAAGATAAGAACGACGAGCAACAACAGGTCGATGAGGAGGAAACAAAG

_

TREU927 (T.b.b) GTGGAAAATGGAAGCAGCGAGGAGGGGTCTTGTTGTGGAAACGAAAGTAACGGTCCCCAT

Eliane (Gp 1 T.b.g) GTGGAAAATGGAAGCAGCGAGGAGGGGTCTTGTTGTGGAAACGAAAGTAACGGTCCCCAT

STIB386A (Gp 2 T.b.g) GTGGAAAATGGAAGCAGCGAGGAGGGGTCTTGTTGTGGAAACGAAAGTAACGGTCCCCAT

STIB386B (Gp 2 T.b.g) GTGGAAAATGGAAGCAGCGAGGAGAGGTCTTGTTGTGGAAACGAAAGTAACGGTCCCCAT

_

TREU927 (T.b.b) GTGATGAAAAAACGTCATGGGGTTGAGGGACCAAGGCCCGTTGACGTGGTTAGTGGTTTC

Eliane (Gp 1 T.b.g) GTGATGAAAAAACGTCATGGGGTTGGGGCACCAAGGCCCGTTGACGTGGTTAGTGGTTTC

STIB386A (Gp 2 T.b.g) GTGATGAAAAAACGTCATGGGGTTGGGGCACCAAGGCCCGTTGACGTGGTTAGTGGTTTC

STIB386B (Gp 2 T.b.g) GTGATGAAAAAACGTCATGGGGTTGAGGGACCAAGGCCCGTTGACGTGGTTAGTGGTTTC

_

TREU927 (T.b.b) CGCAGTTATGCCAGTGCTTCTTTTGCTCTGCTTTCTCTTGTCCGTGTCGGTATGCTCCAG

Eliane (Gp 1 T.b.g) CGCAGTTATGCCAGCGCTTCTTTTGCTCTGCTTTCTCTTGTCCGTGTCGGTATTCTCCAG

STIB386A (Gp 2 T.b.g) CGCAGTTATGCCAGCGCTTCTTTTGCTCTGCTTTCTCTTGTCCGTGTCGGTATGCTCCAG

STIB386B (Gp 2 T.b.g) CGCAGTTATGCCAGCGCTTCTTTTGCTCTGCTTTCTCTTGTCCGTGTCGGTATTCTCCAG

_____<-Gene|

TREU927 (T.b.b) GTGGTGGTGTAGAAAAGACACCGTTTCTTCCAAAGACTGCGCGGGAGTTGCTCCTAAATT

Eliane (Gp 1 T.b.g) GTGGTGGTGTAGAAAAGACACCGTTTCTTCCAAAGACTGCGCGGGAGTTGCTCCTAAATT

STIB386A (Gp 2 T.b.g) GTGGTGGTGTAGAAAAGACACCGTTTCTTCCAAAGACTGCGCGGGAGTTGCTCCTAAATT

STIB386B (Gp 2 T.b.g) GTGGTGGTGTAGAAAAGACACCGTTTCTTCCAAAGACTGCGCGGGAGTTGCTCCCAAATT

TREU927 (T.b.b) GCATTTCCTTCTCTCCTCGTAACATTAAGTGGTCGTGTTGTTGTTTTTTCTTCTTTCCTC

Eliane (Gp 1 T.b.g) GCATTTCCTTCTCTCCTCGTAACATTAAGTGGTCGTTTTGTTGTTTTTTCTTCTTTCCTC

STIB386A (Gp 2 T.b.g) GCATTTCCTTCTCTCCTCGTAACATTAAGTGGTCGTGTTGTTGTTTTTTCTTCTTTCCTC

STIB386B (Gp 2 T.b.g) GCATTTCCTTCTCTCCTCGTAACATTAAGTGGTCGTGTTGTTGTTTTTTCTTCTTTCCTC

TREU927 (T.b.b) TCGCGTGTGGAGGCGAGGCTGAAGACGAACTGCGGCGTCAGACTGCCAAGTGTGAGAAAG

Eliane (Gp 1 T.b.g) TCGCGTGTGGAGGCGAGGCTGAAGACGAACTGCAGCGTCAGACTGCCAAGTGTGAGAAAG

STIB386A (Gp 2 T.b.g) TCGCGTGTGGAGGCGAGGCTGAAGACGAACTGCGGCGTCAGACTGCCAAGTGTGAGAAAG

STIB386B (Gp 2 T.b.g) TCGCGTGTGGAGGCGAGGCTGAAGACGAACTGCAGCGTCAGACTGCCAAGTGTGAGAAAG

TREU927 (T.b.b) AGCGTGAAGTGGTGTGGGGATGATGCCAACGAAGATCTTTTTAATTTTTGGATGTGGGCT

Eliane (Gp 1 T.b.g) AGCGTGAAGTGGTGTGGGGATGATGCCAACGAAGATCTTTTTAATTTTTGGATGTGGGCT

STIB386A (Gp 2 T.b.g) AGCGTGAAGTGGTGTGGGGATGATGCCAACGAAGATCTTTTTAATTTTTGGATGTGGGCT

STIB386B (Gp 2 T.b.g) AGCGTGAAGTGGTGTGGGGATGATGCCAACGAAGATCTTTTTAATTTTTGGATGTGGGCT

TREU927 (T.b.b) TACGTAGTTCGCTGGTAAATATACTTAAGATCTTTCTTTTTTTTTTT--TGTTGTTGTAT

Eliane (Gp 1 T.b.g) TACGTAGTTCGCTGGTAAATATACTTAAGATCTTTCTTTTTTTTTTTGTTGTTGTTGTAT

STIB386A (Gp 2 T.b.g) TACGTAGTTCGCTGGTAAATATACTTAAGATCTTTCTTTTTTTTTTTTTT--TGTTGTAT

STIB386B (Gp 2 T.b.g) TACGTAGTTCGCTGGTAAATATACTTAAGATCTTTCTTTTTTTTTTTGTTGTTGTTGTAT

TREU927 (T.b.b) GTCTTTTTGATTTTGTTGTATTGATTGAATTATTCCCCTTGAGCTTTCTTGTCTTGTACG

Eliane (Gp 1 T.b.g) GTCTTTTTGATTTTGTTGTATTGACTGAATTATTCCCCTTGAGCTTTCTTGTCTTGTACG

STIB386A (Gp 2 T.b.g) GTCTTTTTGATTTTGTTGTATTGACTGAATTATTCCCCTTGAGCTTTCTTGTCTTGTACG

STIB386B (Gp 2 T.b.g) GTCTTTTTGATTTTGTTGTATTGACTGAATTATTCCCCTTGAGCTTTCTTGTCTTGTACG

TREU927 (T.b.b) ATACGTTGGCGAGAAGCTGGTTAAACGAAAACTTTGAAGGAAAAAAATGTGGGATGTGTT

Eliane (Gp 1 T.b.g) ATACGTTGGCGAGAAGCTGGTTAAACGAAAACTTTGAAGGAAAAAAATGTGGGATGTGTT

STIB386A (Gp 2 T.b.g) ATACGTTGGCGAGAAGCTGGTTAAACGAAAACTTTGAAGGAAAAAAATGTGGGATGTGTT

STIB386B (Gp 2 T.b.g) ATACGTTGGCGAGAAGCTGGTTAAACGAAAACTTTGAAGGAAAAAAATGTGGGATGTGTT

TREU927 (T.b.b) TGTGTCTGCTTGTGTGTGTAAACGTAATGGAGGCTTCCCCCCCC------ATTAGTGGCA

Eliane (Gp 1 T.b.g) TGTGTCTGTTTGTGTGTGTAAACGTAATGGAGGCTTCCCCCCCCCCC---ATTAGTGGCA

STIB386A (Gp 2 T.b.g) TGTGTCTGTTTGTGTGTGTAAACGTAATGGAGGCTTCCCCCCCCCCCCCCATTAGTGGCA

STIB386B (Gp 2 T.b.g) TGTGTCTGTTTGTGTGTGTAAACGTAATGGAGGCTTCCCCCCCCCCCCCCATTAGTGGCA

TREU927 (T.b.b) TGCATGCATTTCCGATGCCGACATGCGGTTTTCGGTGAGGGAACAATGCGCCTCACCGTA

Eliane (Gp 1 T.b.g) TGGATGCATTTCCGATGCCGACATGCGGTTTTCGGTGAGGGAACAATGCGCCTCACCGTA

STIB386A (Gp 2 T.b.g) TGCATGCATTTCCGATGCCGACATGCGGTTTTCGGTGAGGGAACAATGCGCCTCACCGTA

STIB386B (Gp 2 T.b.g) TGCATGCATTTCCGATGCCGACATGCGGTTTTCGGTGAGGGAACAATGCGCCTCACCGTA

TREU927 (T.b.b) TCATGTACCGTGAACTACAAGCCACAAAAATATGTGCTTGAATAGTAAGTCCTCCCAACT

Eliane (Gp 1 T.b.g) TCATGTACCGTGAACTACAAGCCACAAAAATATGTGCTTGAATAGTAAGTCCTCCCAACT

STIB386A (Gp 2 T.b.g) TCATGTACCGTGAACTACAAGCCACAAAAATATGTGCTTGAATAGTAAGTCCTCCCAACT

STIB386B (Gp 2 T.b.g) TCATGTACCGTGAACTACAAGCCACAAAAATATGTGCTTGAATAGTAAGTCCTCCCAACT

TREU927 (T.b.b) TCATTTCATGACCAGGAAATTAATCATGTCAAGCCATTGAGCAACTTTTTCTCTTCAGTG

Eliane (Gp 1 T.b.g) TCATTTCATGACCAGGAAATTAATCATGTCAAGCCATTGAGCAACTTTTTCTCTTCAGTG

STIB386A (Gp 2 T.b.g) TCATTTCATGACCAGGAAATTAATCATGTCAAGCCATTGAGCAACTTTTTCTCTTCAGTG

STIB386B (Gp 2 T.b.g) TCATTTCATGACCAGGAAATTAATCATGTCAAGCCATTGAGCAACTTTTTCTCTTCAGTG

TREU927 (T.b.b) GTATGCCTTTGTTCGTTTTCAAAAAAAAAAA--GGAAGAATAAAGAGAAAGGGAGTGGGA

Eliane (Gp 1 T.b.g) GTATGCCTTTGTTCGTTTTCAAAAAAAAAAAAAGGAAGAATAAAGAGAAAGGGAGTGGGA

STIB386A (Gp 2 T.b.g) GTATGCCTTTGTTCGTTTTCAAAAAAAAAAAAAGGAAGAATAAAGAGAAAGGGAGTGGGA

STIB386B (Gp 2 T.b.g) GTATGCCTTTGTTCGTTTTCAAAAAAAAAAAAAGGAAGAATAAAGAGAAAGGGAGTGGGA

TREU927 (T.b.b) AAACTTTTTTTTTTTTGCTACATGTGCCTTTTCTAGTGTGTGCGAGCGCCCGCTGCGAGG

Eliane (Gp 1 T.b.g) AAACTTTTTTTTTT--GCTATATGTGCCTTTTCTAGTGTGTGCGAGTGCCCGCTGCGAGG

STIB386A (Gp 2 T.b.g) AAACTTTTTTTTTTTTGCTATATGCGCCTTTTCTAGTGTGTGCGAGTGCCCGCTGCGAGG

STIB386B (Gp 2 T.b.g) AAACTTTTTTTTTTT-GCTATATGTGCCTTTTCTAGTGTGTGCGAGTGCCCGCTGCGAGG

TREU927 (T.b.b) AATTGAGCTTTCTCCTTTCCTTCCTTTTCCTCCTTTC

Eliane (Gp 1 T.b.g) AATTGAGCTTTCTCCTTTCCTTCCTTTTCCTCCTTTC

STIB386A (Gp 2 T.b.g) AATTGAGCTTTCTCCTTTCCTTCCTTTTCCTCCTTTC

STIB386B (Gp 2 T.b.g) AATTGAGCTTTCTCCTTTCCTTCCTTTTCCTCCTTTC
