# Supplementary figures and images for: Differences between Trypanosoma brucei gambiense Groups 1 and 2 in Their Resistance to Killing by Trypanolytic Factor 1
Source: PLoS Negl Trop Dis. 2011 Sep 6;5(9):e1287. doi: 10.1371/journal.pntd.0001287 (PMC3167774; doi:10.1371/journal.pntd.0001287)

**Figure S4.**


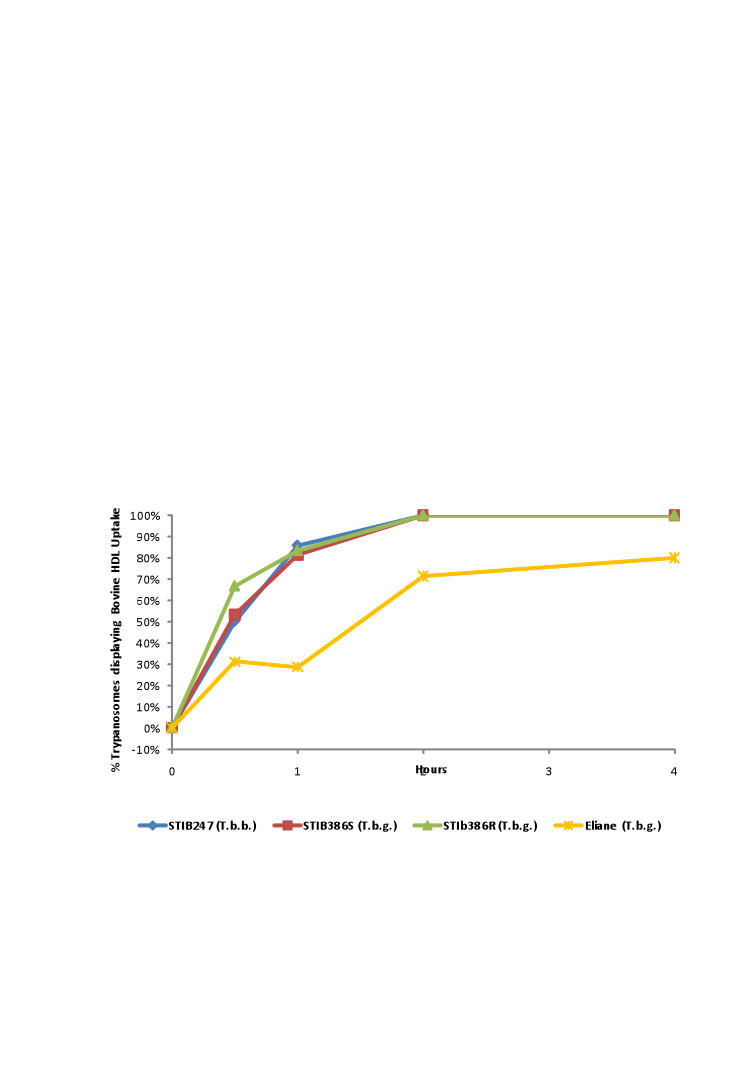

Supplement: Figure S4 — Uptake of bovine HDL of a comparable size to TLF-1, measured by visible concentrations of AlexaFluor® tagged bovine HDL in the parasite body after 4 hour exposure to labelled bovine HDL. (DOC) [file pntd.0001287.s004.doc]
