# Supplementary material for: Differences between Trypanosoma brucei gambiense Groups 1 and 2 in Their Resistance to Killing by Trypanolytic Factor 1
Source: PLoS Negl Trop Dis. 2011 Sep 6;5(9):e1287. doi: 10.1371/journal.pntd.0001287 (PMC3167774; doi:10.1371/journal.pntd.0001287)
Supplement: Figure S5 — Cell counts of viable motile cells measured after 24-hour exposure to various concentrations of recombinant APOL1 for the T. b. brucei strain STIB247, the resistant and sensitive isogenic lines of the group 2 T. b. gambiense STIB386 and the group 1 T. b. gambiense ELIANE. Standard error is indicated (n = 2). (DOC) [file pntd.0001287.s005.doc]

**Figure S5.**
